# SETD2 regulates chromatin accessibility and transcription to suppress lung tumorigenesis

**DOI:** 10.1172/jci.insight.154120

**Published:** 2023-02-22

**Authors:** Yuchen Xie, Merve Sahin, Toru Wakamatsu, Akane Inoue-Yamauchi, Wanming Zhao, Song Han, Amrita M. Nargund, Shaoyuan Yang, Yang Lyu, James J. Hsieh, Christina S. Leslie, Emily H. Cheng

**Affiliations:** 1 Human Oncology and Pathogenesis Program, Memorial Sloan Kettering Cancer Center (MSKCC), New York, New York, USA.; 2 Gerstner Sloan Kettering Graduate School of Biomedical Sciences, New York, New York, USA.; 3 Computational and Systems Biology Program, MSKCC, New York, New York, USA.; 4 Tri-Institutional Training Program in Computational Biology and Medicine, New York, New York, USA.; 5 Molecular Oncology, Department of Medicine, Washington University, St. Louis, Missouri, USA.; 6 Department of Pathology and Laboratory Medicine, MSKCC, New York, New York, USA.; 7 Weill Cornell Medical College, New York, New York, USA.

**Keywords:** Oncology, Epigenetics, Lung cancer

## Abstract

*SETD2*, a H3K36 trimethyltransferase, is the most frequently mutated epigenetic modifier in lung adenocarcinoma, with a mutation frequency of approximately 9%. However, how SETD2 loss of function promotes tumorigenesis remains unclear. Using conditional *Setd2*-KO mice, we demonstrated that *Setd2* deficiency accelerated the initiation of *Kras^G12D^*-driven lung tumorigenesis, increased tumor burden, and significantly reduced mouse survival. An integrated chromatin accessibility and transcriptome analysis revealed a potentially novel tumor suppressor model of SETD2 in which SETD2 loss activates intronic enhancers to drive oncogenic transcriptional output, including the KRAS transcriptional signature and PRC2-repressed targets, through regulation of chromatin accessibility and histone chaperone recruitment. Importantly, SETD2 loss sensitized KRAS-mutant lung cancer to inhibition of histone chaperones, the FACT complex, or transcriptional elongation both in vitro and in vivo. Overall, our studies not only provide insight into how SETD2 loss shapes the epigenetic and transcriptional landscape to promote tumorigenesis, but they also identify potential therapeutic strategies for *SETD2* mutant cancers.

## Graphical abstract

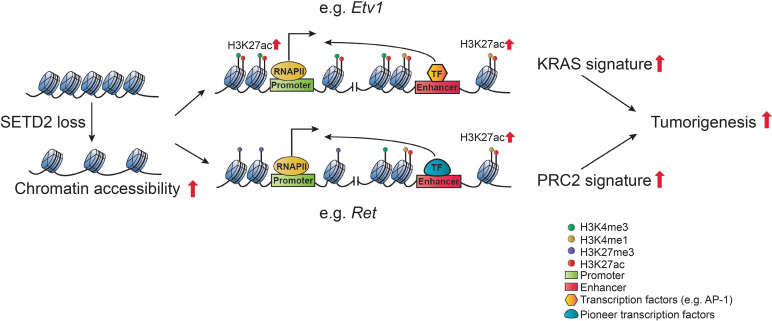

## Introduction

SETD2 is an RNA polymerase II–associated (Pol II–associated) histone methyltransferase involved in the cotranscriptional methylation of H3K36 to generate H3K36me3 in the bodies of actively transcribed genes (1–5), and this process is important for transcriptional elongation, repression of cryptic transcription initiation, cotranscriptional RNA processing, and alternative splicing (1–7). In addition, SETD2-mediated H3K36me3 has been shown to be involved in DNA mismatch repair (8), DNA double-strand break repair by homologous recombination, and the maintenance of genome stability (9, 10). *SETD2* is one of the most frequently mutated chromatin-modifying genes across different cancer types, with the highest mutation rate in clear cell renal cell carcinoma (ccRCC, 13%) followed by lung adenocarcinoma (9%) (11, 12). Based on The Cancer Genome Atlas Lung Adenocarcinoma (TCGA-LUAD) data set, *SETD2* is the eighth most commonly mutated gene and the most frequently mutated epigenetic modifier in lung adenocarcinoma (12). The majority of *SETD2* mutations identified in lung adenocarcinoma are truncating mutations that result in the production of truncated proteins lacking either the histone methyltransferase Su(var)3-9, Enhancer-of-zeste and Trithorax (SET) domain or the Set2-Rpb1–interacting (SRI) domain that mediates the interaction of SETD2 with Pol II. Furthermore, loss of heterozygosity (LOH) at chromosome 3p, where *SETD2* resides, is commonly detected in lung adenocarcinoma (12–15). Together, these data support that *SETD2* is a tumor-suppressor gene in lung adenocarcinoma. Notably, *SETD2* mutations often cooccur with other well-established driver mutations, such as *KRAS*, *EGFR*, and *BRAF*, that activate the RTK/RAS/RAF pathway in lung adenocarcinoma (12), suggesting that SETD2 inactivation probably cooperates with these driver mutations to promote lung tumorigenesis. *Setd2* deficiency was recently reported to cooperate with *Kras^G12D^* or both *Kras^G12D^* and *p53* deficiency to promote the initiation of mouse lung cancer using CRISPR/Cas9-mediated genome editing (16, 17). Furthermore, *Setd2* loss was reported to promote Kras-induced acinar-to-ductal metaplasia and epithelia-mesenchymal transition during pancreatic carcinogenesis (18). Nonetheless, how SETD2 loss-of-function promotes tumorigenesis in lung remains unclear.

Here, we have generated conditional *Setd2*-KO mice to interrogate the molecular mechanisms by which *Setd2* deficiency cooperates with *Kras^G12D^* to promote lung tumor initiation. Of note, homozygous deletion of *Setd2* in mice results in embryonic lethality, vascular defects, and loss of H3K36me3 without alterations of H3K36me1 and H3K36me2 (4). Consistent with the reported findings (16), we showed that *Setd2* deficiency accelerated the initiation of *Kras^G12D^*-driven lung tumorigenesis, increased tumor burden, and significantly reduced mouse survival. Mechanistically, we demonstrated that *Setd2* deficiency resulted in a coordinated reprogramming of the epigenome and the transcriptome, which enables the amplification of specific oncogenic signatures that promote *Kras^G12D^*-driven lung tumorigenesis. SETD2 loss appears to create a permissive epigenetic landscape for the cooperating driver oncogenes to amplify their transcriptional output for tumor initiation in a context-dependent manner. Furthermore, we uncovered mechanism-based therapeutic strategies for SETD2-deficient cancers through inhibition of histone chaperones and transcription elongation.

## Results

### Setd2 deficiency cooperates with Kras^G12D^ to promote lung tumorigenesis.

**Figure 1:**
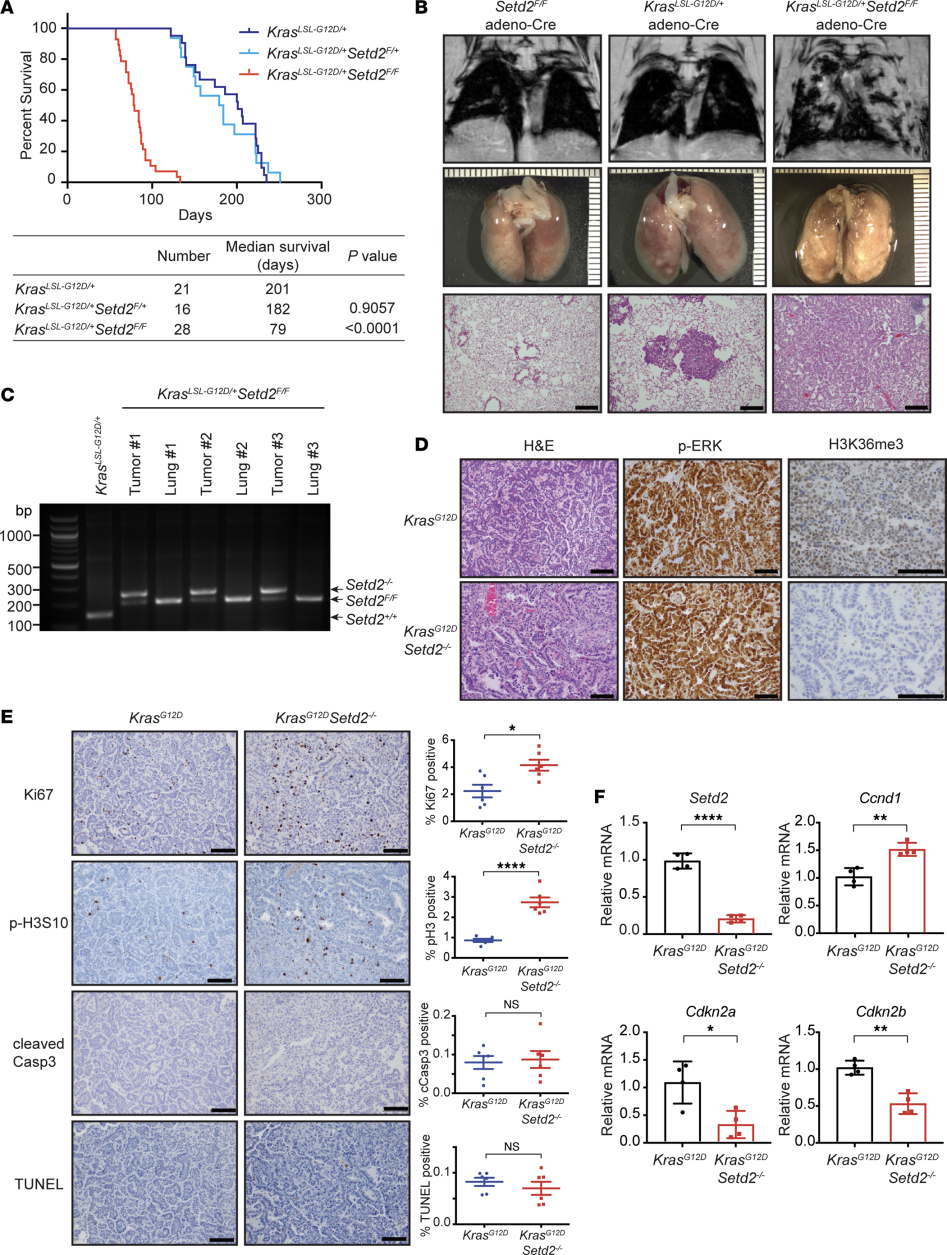
*Setd2* deficiency cooperates with *Kras^G12D^* to promote lung tumorigenesis. (**A**) Kaplan-Meier survival curves of *Kras^LSL–G12D/+^*, *Kras^LSL–G12D/+^ Setd2^fl/+^*, and *Kras^LSL–G12D/+^ Setd2^fl/fl^* mice after adeno-Cre infection. *P* values denote the comparison of mice of the indicated genotypes with *Kras^LSL–G12D/+^* mice (Mantel–Cox test). (**B**) Representative MRI, gross images, and histological sections stained with H&E of lungs from *Setd2^fl/fl^* mice at 1 year, *Kras^LSL–G12D/+^* mice at 3 months, and *Kras^LSL–G12D/+^ Setd2^fl/fl^* mice at 3 months after adeno-Cre infection. Scale bars: 200 μm. (**C**) PCR-based genotyping of *Setd2* alleles in lung tumors from a representative *Kras^LSL–G12D/+^* mouse and in lung tumors and adjacent normal lung tissues from representative *Kras^LSL–G12D/+^ Setd2^fl/fl^* mice after adeno-Cre infection. (**D**) Representative H&E staining and IHC for phospho-ERK and H3K36me3 of *Kras^G12D^* and *Kras^G12D^Setd2^–/–^* lung tumors. Scale bars: 100 μm. (**E**) Representative IHC for Ki67, phospho-H3S10, cleaved caspase-3, and TUNEL assays of *Kras^G12D^* and *Kras^G12D^Setd2^–/–^* lung tumors. The percentage of positive cells for each staining was quantified (mean ± SD, *n* = 6). Scale bars: 100 μm. (**F**) The mRNA levels of *Setd2*, *Ccnd1*, *Cdkn2a*, and *Cdkn2b* were assessed by qPCR. Data were normalized against β*-*actin (mean ± SD, *n* = 4). *P < 0.05; **P < 0.01; ****P < 0.0001 by Student’s *t* test.

To investigate the oncogenic cooperation between SETD2 loss and RAS activation in lung cancer pathogenesis in a whole-organism setting, we have generated conditional *Setd2*-KO mice (Supplemental Figure 1A; supplemental material available online with this article; https://doi.org/10.1172/jci.insight.154120DS1). *Setd2^fl/fl^* mice were crossed to mice carrying a conditionally activatable *Lox-Stop-Lox Kras^G12D^* allele (hereafter called *Kras^LSL–G12D^*) (19). Intranasal administration of Cre-expressing adenovirus (adeno-Cre) was performed to activate the expression of *Kras^G12D^* as well as to delete the floxed *Setd2* alleles. As reported in ref. 19, *Kras^LSL–G12D/+^* mice following adeno-Cre administration developed adenoma and nonmetastatic adenocarcinoma with a median survival of 201 days. Strikingly, homozygous deletion of *Setd2* accelerated the initiation of *Kras^G12D^* lung tumors, increased tumor burden, and significantly reduced mouse survival (Figure 1, A and B, and Supplemental Figure 1, B and C). The majority of *Kras^LSL–G12D/+^ Setd2^fl/fl^* mice died from lung adenocarcinoma within 3 months following adeno-Cre infection (median survival, 79 days), whereas *Kras^LSL–G12D/+^ Setd2^fl/+^* mice exhibited comparable survival to *Kras^LSL–G12D/+^* mice (Figure 1A). Notably, all *Setd2^fl/fl^* mice remained healthy at 1 year after adeno-Cre infection (Figure 1B), indicating that *Setd2* deficiency alone is insufficient for tumor initiation. PCR-based genotyping confirmed the efficient deletion of floxed *Setd2* alleles in lung tumors, with greatly reduced *Setd2* expression determined by quantitative PCR (qPCR) (Figure 1, C and F).

Histological examination of *Kras^G12D^Setd2^–/–^* lung tumors showed mostly well differentiated to moderately differentiated adenocarcinoma with focal invasion and juxtatumoral desmoplastic stromal reaction (Figure 1B and Supplemental Figure 1D). Although *Kras^LSL–G12D/+^* mice only displayed small adenomas at 3 months, some mice developed extensive adenocarcinoma at 5–6 months following adeno-Cre infection, as reported (19). For the ensuing molecular characterization, we used *Kras^G12D^Setd2^–/–^* lung tumors at 3 months following adeno-Cre infection and *Kras^G12D^* lung tumors at 5–6 months following adeno-Cre infection that exhibited comparable tumor grades (Supplemental Figure 1E). IHC showed that *Kras^G12D^Setd2^–/–^* lung tumors exhibited reduced H3K36me3 and yet comparable phospho-ERK staining in comparison with *Kras^G12D^* lung tumors (Figure 1D). *Kras^G12D^Setd2^–/–^* lung tumors showed increased Ki67 and phospho-H3S10 staining, with no differences in cell death markers in comparison with *Kras^G12D^* lung tumors (Figure 1E). Consistent with increased proliferation markers in *Kras^G12D^Setd2^–/–^* lung tumors, *Setd2* deficiency upregulated *cyclin D1* and downregulated *Cdkn2a* and *Cdkn2b* (Figure 1F). Collectively, our studies presented compelling evidence of the oncogenic cooperation between *Setd2* deficiency and *Kras^G12D^* in accelerating lung tumorigenesis.

### Setd2 deficiency increases chromatin accessibility and oncogenic transcriptional output in Kras^G12D^ lung tumors.

**Figure 2:**
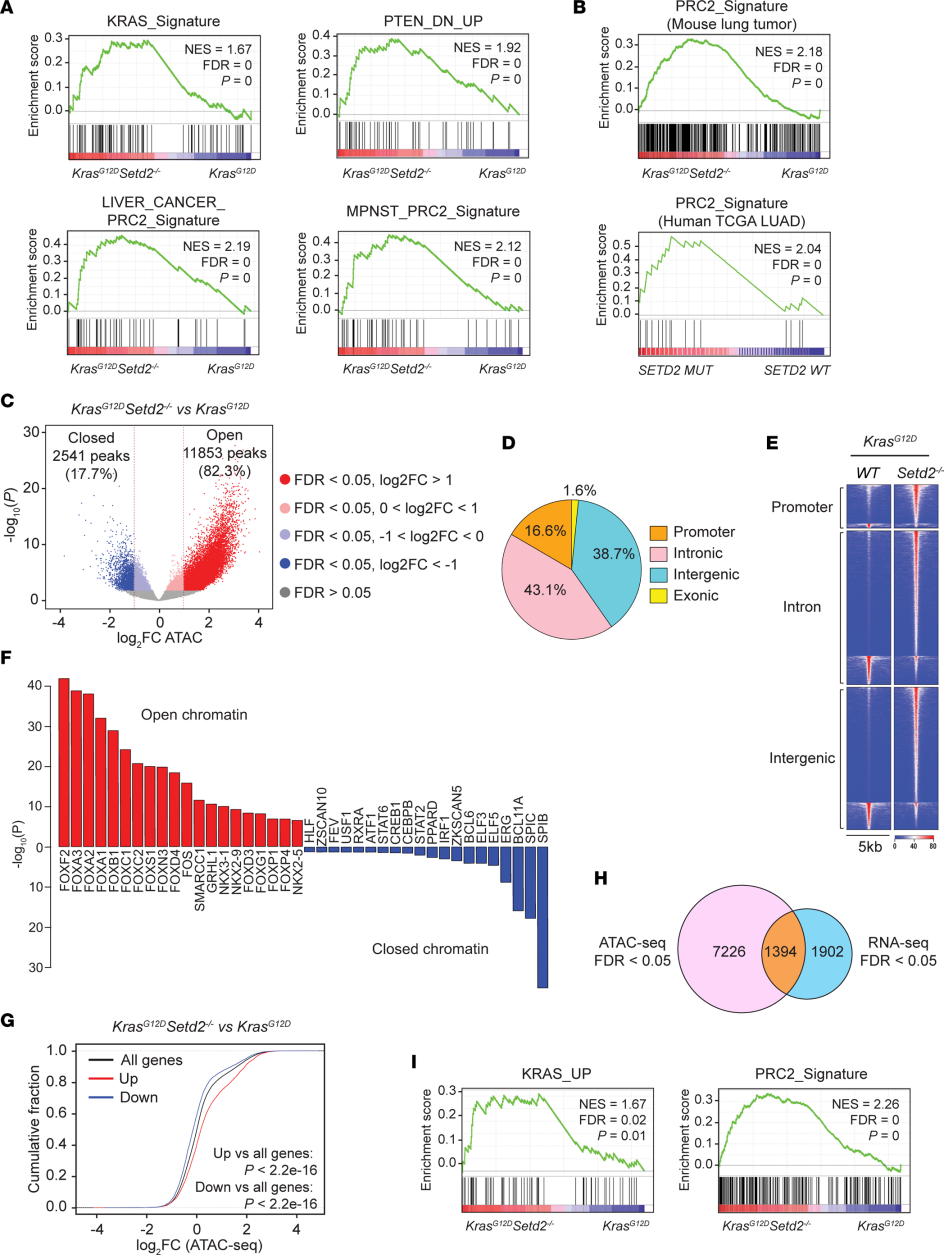
*Setd2-*deficient *Kras^G12D^* lung tumors show increased chromatin accessibility and oncogenic transcriptional output compared with *Kras^G12D^* lung tumors. (**A**) GSEA plots of the differentially expressed genes (FDR < 0.05) comparing *Kras^G12D^Setd2^–/–^* with *Kras^G12D^* mouse lung tumors using the indicated gene sets. MPNST, malignant peripheral nerve sheath tumors. NES, normalized enrichment score. (**B**) PRC2 signature enrichment plots of the differentially expressed genes (FDR < 0.05) comparing *Kras^G12D^Setd2^–/–^* with *Kras^G12D^* mouse lung tumors and comparing *SETD2^MT^* with *SETD2*^WT^ human lung adenocarcinomas (LUAD) from TCGA using the composite PRC2 signature. (**C**) Volcano plots of ATAC-Seq peaks comparing dissociated *Kras^G12D^Setd2^–/–^* with *Kras^G12D^* mouse lung tumor cells. The number of peaks with significant changes (FDR < 0.05 and log_2_FC > 1) upon *Setd2* deletion is shown. (**D**) Pie chart showing the percentage of differentially accessible ATAC-Seq peaks (FDR < 0.05) at promoter, intronic, intergenic, and exonic regions comparing *Kras^G12D^Setd2^–/–^* with *Kras^G12D^* mouse lung tumors. (**E**) Heatmap of differentially accessible ATAC-Seq peaks described in **C** (FDR < 0.05 and log_2_FC > 1) in 5 kb window grouped by localization at promoter, intron, and intergenic regions. (**F**) The 20 most significantly enriched transcription factor binding motifs in open (red) and closed (blue) chromatin peaks comparing *Kras^G12D^Setd2^–/–^* with *Kras^G12D^* mouse lung tumors. (**G**) Distribution of chromatin accessibility changes associated with significantly upregulated (red) or downregulated (blue) genes comparing *Kras^G12D^Setd2^–/–^* with *Kras^G12D^* mouse lung tumors. *P* values calculated using 1-sided Kolmogorov-Smirnov (KS) test comparing peaks associated with differentially expressed genes to all genes. (**H**) Venn diagram showing overlap of differentially expressed genes detected by RNA-Seq (FDR < 0.05) and genes with differentially accessible ATAC-Seq peaks (FDR < 0.05) comparing *Kras^G12D^Setd2^–/–^* with *Kras^G12D^* mouse lung tumors. (**I**) GSEA plots of the 1,394 differentially expressed genes shown in **H** using the KRAS and PRC2 signatures.

To investigate the impact of SETD2 loss-induced transcriptome changes on the pathogenesis of *Kras^G12D^*-driven lung cancer, RNA-Seq was performed on *Kras^G12D^* and *Kras^G12D^Setd2^–/–^* lung tumors with comparable histopathological features and tumor grades (Supplemental Figure 1E). *Setd2* deficiency in *Kras^G12D^* lung tumors led to a global alteration of transcriptome with 3,296 differentially expressed genes (FDR < 0.05; Supplemental Figure 2A). Gene set enrichment analysis (GSEA) revealed upregulation of several oncogenic pathways upon SETD2 loss, including the KRAS transcriptional signature, the PTEN-loss transcriptional signature, and PRC2-repressed targets identified in liver cancer (20), and malignant peripheral nerve sheath tumors (MPNST) (21) (Figure 2A and Supplemental Table 1). Although SETD2 loss does not affect the KRAS/RAF/MEK/ERK signal transduction pathway (Figure 1D), it enhances the transcriptional output downstream of KRAS signaling (Figure 2A). SETD2 loss also led to upregulation of G-protein–coupled receptor signaling, DNA packaging, and RNA catabolism (Supplemental Table 1).

In mammalian cells, 2 main polycomb-repressive complexes (PRCs) have been defined: PRC1 and PRC2, both of which repress gene expression (22, 23). The PRC2 complex catalyzes the methylation of histone H3 at lysine 27 through its enzymatic subunits EZH1 and EZH2; the enrichment of H3K27me3 correlates with gene silencing (22, 23). Of note, PRC2 exerts either oncogenic or tumor-suppressive function in a context-dependent manner. Loss of PRC2 complex has been reported to promote tumor aggressiveness in *p53*-deficient *Kras^G12D^*-driven mouse lung cancer (24). Our demonstration of upregulation of PRC2-suppressed target genes in response to *Setd2* deletion is functionally equivalent to the inactivation of the PRC2 complex, which probably contributes to lung tumor progression. By merging various reported PRC2 modules (20, 21, 25–27), we generated a composite PRC2 signature detailed in the method section. Remarkably, both mouse lung tumors and human lung adenocarcinomas from TCGA data set showed highly enriched PRC2 signature in tumors with SETD2 loss (Figure 2B). Of note, no global difference in H3K27me3 levels was observed comparing *Kras^G12D^Setd2^–/–^* with *Kras^G12D^* tumors (Supplemental Figure 2B), suggesting that SETD2 loss–induced upregulation of PRC2 targets does not simply occur through a direct inactivation of the PRC2 complex. In addition, no global difference in H3K4me3, H3K4me1, or K3K27ac levels was observed comparing *Kras^G12D^Setd2^–/–^* with *Kras^G12D^* tumors (Supplemental Figure 2C).

We hypothesized that increased transcriptional output of oncogenic pathways in *Setd2*-deficient lung tumors may be caused by an altered epigenetic landscape upon the ablation of H3K36me3 marks. To interrogate this hypothesis, ATAC-Seq (an assay for transposase-accessible chromatin using sequencing) was performed on dissociated mouse lung tumor cells to assess genome-wide changes in chromatin accessibility (28). *Setd2* deletion in *Kras^G12D^* lung tumors induced significant genome-wide chromatin accessibility changes at ~14,400 sites, among which 82.3% showed increased chromatin accessibility (Figure 2C and Supplemental Figure 2D). Among the differentially accessible ATAC-Seq peaks (FDR < 0.05), 43.1% were found at introns, 38.7% at intergenic regions, 16.6% at promoters, and 1.6% at exons (Figure 2, D and E). A genome-wide increase in chromatin accessibility was also observed in SETD2-deficient human ccRCC and primary mouse renal tubular epithelial cells compared with respective SETD2-proficient counterparts (29), indicating that increased chromatin accessibility is a primary phenotype caused by SETD2 loss across different tissue types. This is consistent with the reported association of *SETD2* mutations with increased chromatin accessibility preferentially in gene bodies in human ccRCC tumors (30).

The genomic loci with open chromatin peaks induced by *Setd2* deletion in *Kras^G12D^* lung tumors were highly enriched with Forkhead box (FOX) family transcription factor binding motifs (TFBM) (Figure 2F). The open chromatin peaks were also enriched with the binding motif of FOS, one of the key transcription factors downstream of ERK signaling that drives RAS-mediated transcription (31). This is consistent with the enhanced KRAS signature observed in *Kras^G12D^Setd2^–/–^* lung tumors (Figure 2A). Notably, Gene Ontology (GO) and KEGG pathway analysis of genes with open chromatin peaks upon *Setd2* deletion also showed enrichment of the RAS signaling pathway (Supplemental Figure 2E). These findings prompted us to investigate the correlation between SETD2 loss-induced alterations in chromatin accessibility and transcriptional output by integrating the ATAC-Seq data and the RNA-Seq data. Overall, the upregulated genes upon *Setd2* deletion in *Kras^G12D^* lung tumors exhibited more open chromatin, whereas the downregulated genes exhibited more closed chromatin (Figure 2G, *P* < 2.2 ***×*** 10^–16^). GSEA of the differentially expressed genes detected by RNA-Seq that also exhibited differentially accessible ATAC-Seq peaks showed upregulation of KRAS and PRC2 signatures upon SETD2 loss (Figure 2, H and I). Consistently, the genes that were most upregulated in response to SETD2 loss within the KRAS or PRC2 signature displayed mainly open chromatin status, whereas those that were most downregulated displayed closed chromatin status (Supplemental Figure 2F). Collectively, our data reveal that SETD2 loss increases chromatin accessibility to enhance the oncogenic transcriptional output.

### SETD2 loss induces ETV1 expression through activation of an intronic enhancer to promote transformation.

**Figure 3:**
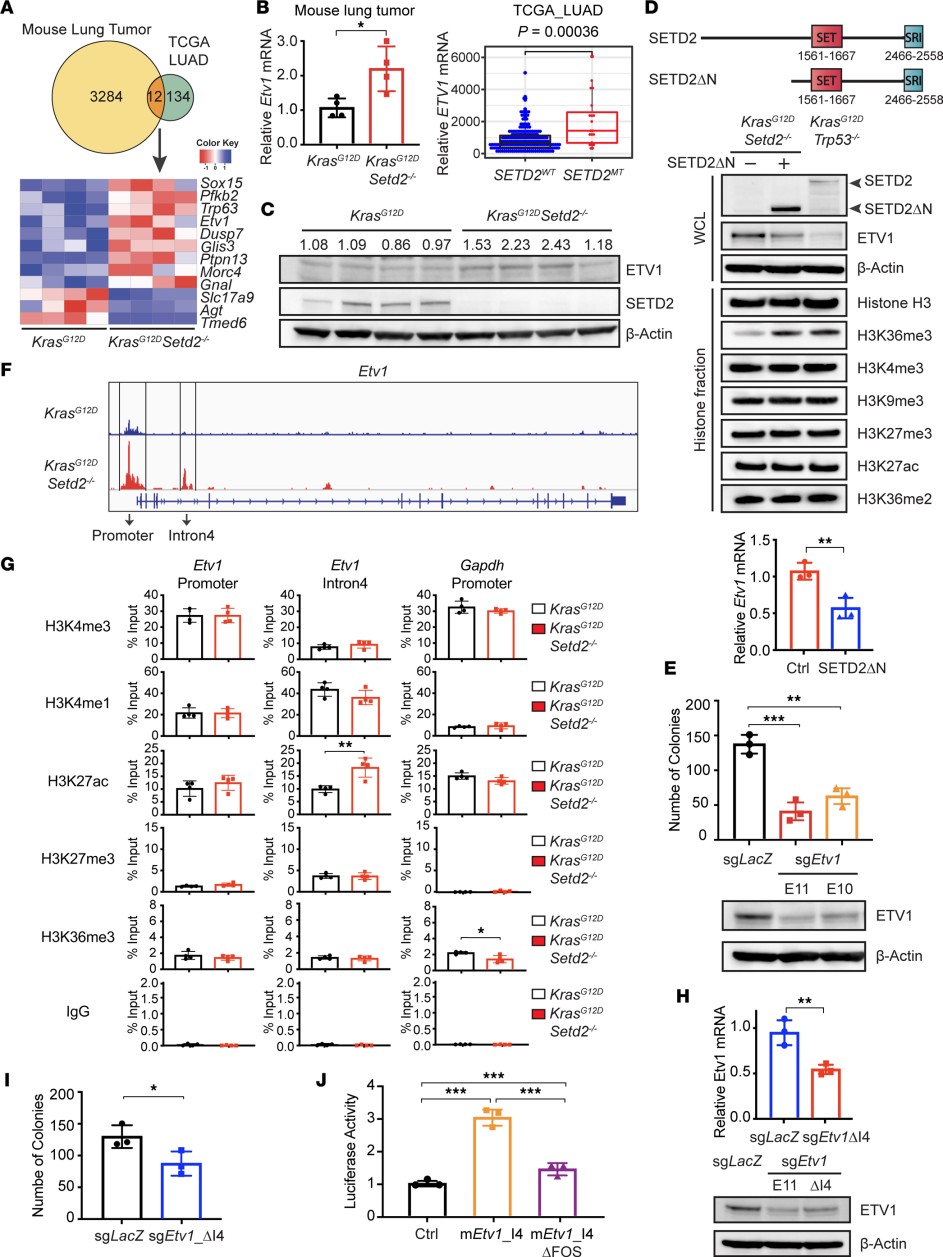
*Setd2* deficiency induces *Etv1* through activation of an intronic enhancer to promote transformation. (**A**) Venn diagram showing overlap of differentially expressed genes (FDR < 0.05) comparing *Kras^G12D^Setd2^–/–^* with *Kras^G12D^* mouse lung tumors and comparing *SETD2^MT^* (*n* = 20) with *SETD2*^WT^ (*n* = 210) human lung adenocarcinomas from TCGA. Heatmap showing these genes in mouse lung tumors. (**B**) Left, qPCR analysis of *Etv1* in mouse lung tumors (mean ± SD, *n* = 4). Right, the normalized *ETV1* expression comparing *SETD2^MT^* with *SETD2*^WT^ human lung adenocarcinomas was obtained from cBioPortal. (**C**) Immunoblot analyses of the indicated mouse lung tumors. The number denotes the ETV1 expression normalized against β-actin (*P* = 0.0301, *Kras^G12D^* versus *Kras^G12D^Setd2^–/–^*). (**D**) A schematic of the domain structure of SETD2 and SETD2ΔN. Whole cell lysates (WCL) and histone fractions from primary *Kras^G12D^Setd2^–/–^* (KS) mouse lung tumor cells ± SETD2ΔN transduction or *Kras^G12D^p53^–/–^* mouse lung tumor cells were analyzed by immunoblots. The *Etv1* mRNA levels were assessed by qPCR (mean ± SD, *n* = 3). (**E**) Primary KS cells transduced with the indicated sgRNAs were analyzed by soft agar colony formation assays and immunoblots. (**F**) Representative ATAC-Seq tracks at the *Etv1* locus in mouse lung tumors. (**G**) Primary mouse lung tumor cells were assessed by ChIP-qPCR at the indicated genomic regions (mean ± SD, *n* = 3). (**H** and **I**) KS cells transduced the indicated sgRNAs were analyzed by qPCR (mean ± SD, *n* = 3), immunoblots, or soft agar colony formation assays. (**J**) A549 cells were transiently transfected with pGL2-pro vector or pGL2-pro containing the putative intron 4 enhancer of *Etv1* ± deletion of the FOS binding motif, together with the pRL-SV40 plasmid (Promega) as a normalization control (mean ± SD, *n* = 3). *P < 0.05; **P < 0.01; ***P < 0.001 by Student’s *t* test.

To understand how *Setd2* deficiency increases chromatin accessibility to induce the expression of KRAS signature, we first focused on *Etv1*, one of the transcription factors downstream of ERK signaling and a well-defined oncogene in multiple cancer types (31, 32). Notably, *Etv1* is among the 12 genes that are differentially expressed upon SETD2 loss in both mouse and human lung adenocarcinomas (Figure 3A). We first confirmed that both *Etv1* mRNA and ETV1 protein were upregulated in *Kras^G12D^Setd2^–/–^* mouse lung tumors compared with *Kras^G12D^* tumors (Figure 3, B and C). Due to the large coding sequence of *SETD2*, efficient transduction of the full-length SETD2 using a retroviral or lentiviral vector was not possible. Nevertheless, it has been reported that the N-terminal truncated SETD2 (SETD2ΔN) is fully functional (33). Consistently, we demonstrated that retroviral transduction of the SETD2ΔN lacking the first 1,241 amino acids while retaining all the important functional domains was sufficient to fully restore H3K36me3 and reduce *Etv1* mRNA and protein in primary cells derived from *Kras^G12D^Setd2^–/–^* mouse lung tumors (Figure 3D). Functionally, lentiviral transduction of Cas9 and the sgRNA targeting *Etv1* significantly reduced the ETV1 protein expression and the ability of *Kras^G12D^Setd2^–/–^* mouse lung tumor cells to form colonies in soft agar (Figure 3E). Overall, these data suggest that ETV1 is one of the important downstream oncogenic targets induced upon SETD2 loss to promote oncogenic transformation.

To interrogate how chromatin accessibility might affect *Etv1* expression in lung cancer, we assessed the ATAC-Seq tracks at the *Etv1* locus, which revealed significantly increased chromatin accessibility at both promoter and intron 4 upon SETD2 loss (Figure 3F). Notably, the distinct ATAC-Seq peak at the intron 4 of mouse *Etv1* coincided with H3K4me1 ChIP-Seq peaks shown in the mouse lung tissue ENCODE data (Supplemental Figure 3A), and this distinct peak likely represents an intronic enhancer. H3K4me1 and H3K27ac are commonly used to annotate enhancers, and H3K27ac specifically marks active enhancers (34–36). Accordingly, we hypothesized that SETD2 loss may increase chromatin accessibility of oncogenic genes to transcription factors and chromatin modifiers — e.g., AP-1 (a dimeric complex composed of members from the JUN, FOS, or ATF protein families — which in turn increases H3K27ac levels and activates certain intronic enhancers to drive respective gene expression. To examine this hypothesis, ChIP-qPCR was performed on dissociated mouse lung tumor cells to assess the impact of *Setd2* deletion in chromatin modifications within the *Etv1* locus. *Setd2* deficiency significantly increased H3K27ac at the intron 4 of *Etv1* (Figure 3G), suggesting that SETD2 loss activates this intronic enhancer. To demonstrate direct regulation of *Etv1* expression by this putative intronic enhancer, CRISPR/Cas9-mediated deletion of the ATAC-Seq peak region at the intron 4 of *Etv1* was performed, which led to reduced *Etv1* expression and soft agar colony formation of *Kras^G12D^Setd2^–/–^* mouse lung tumor cells (Figure 3, H and I, and Supplemental Figure 3, B and C). To further prove the presence of enhancer activity at mouse *Etv1* intron 4, we cloned the DNA fragment from the ATAC-Seq peak region into a luciferase reporter construct that was subsequently transfected into A549, a human *KRAS* mutant lung cancer cell line. Indeed, this DNA fragment conferred a ~3-fold increase in luciferase activity (Figure 3J). Because motif analysis identified a FOS binding site within the intron 4 enhancer of *Etv1* (Supplemental Figure 3D), we next assessed the potential contribution of FOS binding to enhancer activation by deleting the FOS binding motif in the luciferase reporter construct. Deletion of the FOS binding motif diminished the ability of the *Etv1* intron 4 enhancer to induce luciferase activity (Figure 3J). Collectively, these data support that SETD2 loss creates an epigenetic landscape consisting of open chromatin, enabling transcription factors and chromatin modifiers to activate intronic enhancers and amplify the KRAS-driven transcriptional output.

**Figure 4:**
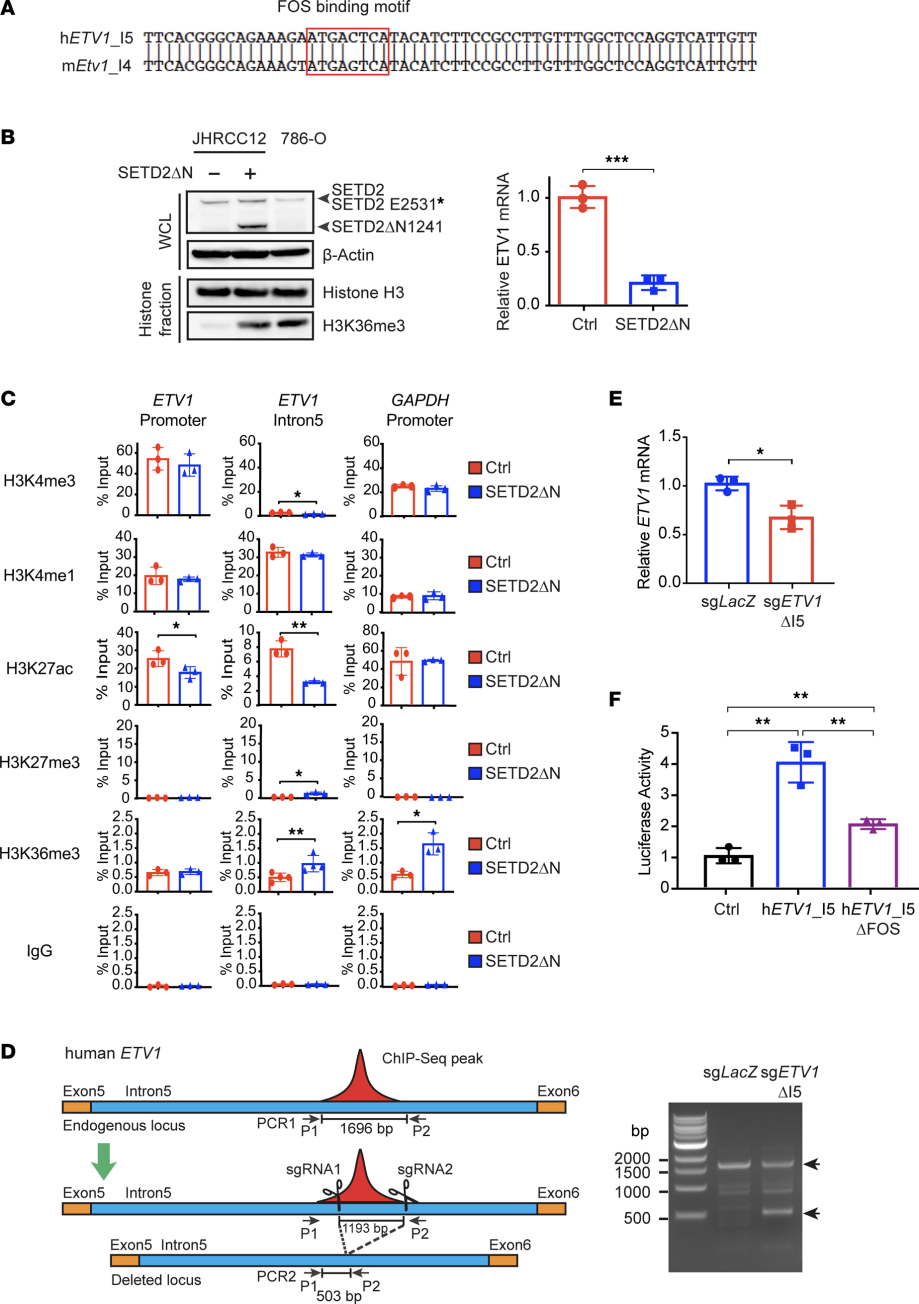
SETD2 loss induces human *ETV1* expression through activation of an intronic enhancer. (**A**) Sequence homology between ATAC-Seq peak regions at the intron 4 of mouse *Etv1* and the intron 5 of human *ETV1*. (**B**) Whole cell lysates (WCL) and histone fractions from JHRCC12 cells infected with control retrovirus or retrovirus expressing SETD2ΔN or from 786-O cells were analyzed by immunoblots. The mRNA levels of *ETV1* were assessed in the indicated JHRCC12 cells by qPCR and normalized against β-actin (mean ± SD, *n* = 3). (**C**) JHRCC12 cells infected with control retrovirus or retrovirus expressing SETD2ΔN were assessed by ChIP-qPCR using the indicated antibodies for the promoter and intron 5 of *ETV1* and the promoter of *GAPDH*. Data shown are the percent input (mean ± SD, *n* = 3). (**D**) A schematic of the strategy used to delete the conserved region (1,193 bp) at the intron 5 (9,157 bp) of *ETV1* in JHRCC12 cells using CRISPR/Cas9-mediated genome editing. The positions of primers (P1 and P2) used for PCR-based validation of genome editing are indicated. PCR-based genotyping using the P1 and P2 primers was performed on JHRCC12 cells ± CRISPR/Cas9-mediated deletion of the intron 5 of *ETV1*. (**E**) The mRNA levels of *ETV1* in JHRCC12 cells infected with lentivirus expressing the indicated sgRNAs were assessed by qPCR and normalized against β*-*actin (mean ± SD, *n* = 3). (**F**) A549 cells were transiently transfected with pGL2-pro vector or pGL2-pro containing the putative intron 5 enhancer of *ETV1* without or with deletion of the FOS binding motif together with the pRL-SV40 plasmid (Promega) as a normalization control. The firefly and Renilla luciferase activities were assessed and normalized (mean ± SD, *n* = 3). *P < 0.05; **P < 0.01; ***P < 0.001 by Student’s *t* test.

We next investigated whether SETD2-mediated regulation of ETV1 is conserved in human ccRCC in which the highest mutation rate of *SETD2* is observed (11). The distinct ATAC-Seq peak at the intron 4 of mouse *Etv1* coincided with H3K4me1 ChIP-Seq peaks at the equivalent intron 5 of human *ETV1* with significant sequence homology (Figure 4A and ENCODE data not shown). To determine whether the conserved sequence at the intron 5 of human *ETV1* also contains an enhancer that is regulated by SETD2, ChIP-qPCR was performed on a patient-derived ccRCC cell line JHRCC12 that harbors a truncating mutation of *SETD2* at the SRI domain (p.E2531*) (37). Retroviral transduction of SETD2ΔN in JHRCC12 restored H3K36me3 to levels comparable with 786-O cells carrying WT *SETD2*, and it reduced *ETV1* expression (Figure 4B). ChIP-qPCR showed that transduction of SETD2ΔN in JHRCC12 cells greatly reduced H3K27ac at the intron 5 of *ETV1* (Figure 4C), suggesting that SETD2 loss of function activates this intronic enhancer. Transduction of SETD2ΔN in JHRCC12 cells also reduced H3K27ac at the promoter of *ETV1* but to a lesser extent than the intron 5 (Figure 4C). Importantly, CRISPR/Cas9-mediated deletion of the intron 5 enhancer significantly reduced *ETV1* expression in JHRCC12 cells (Figure 4, D and E), supporting a direct activation of *ETV1* transcription by this intronic enhancer. Furthermore, luciferase reporter assays confirmed the presence of enhancer activity within this putative enhancer in human *ETV1*, and the enhancer activity was abrogated by deletion of the FOS-binding motif (Figure 4F). Collectively, data obtained from both mouse lung tumors and human ccRCC revealed a conserved regulatory mechanism of *ETV1* transcription upon SETD2 loss through increased chromatin accessibility and intronic enhancer activity.

### Setd2 deficiency increases chromatin accessibility and activates enhancers to induce KRAS and PRC2 signature genes.

**Figure 5:**
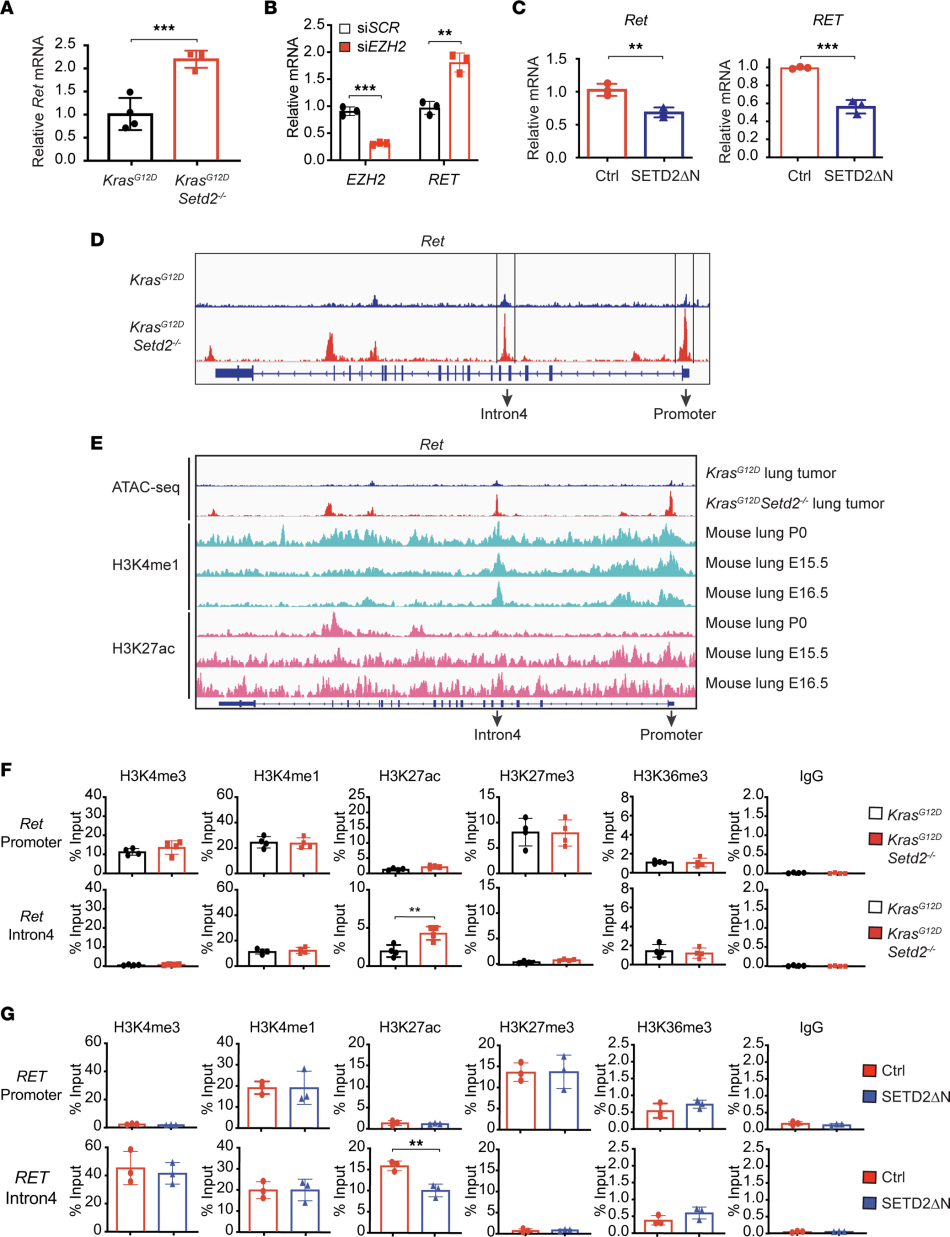
*Setd2* deficiency increases chromatin accessibility and activates enhancers to induce PRC2 and KRAS signature genes. (**A**) The mRNA levels of *Ret* in *Kras^G12D^* and *Kras^G12D^Setd2^–/–^* mouse lung tumors were assessed by qPCR and normalized against β*-*actin (mean ± SD, *n* = 4). (**B**) JHRCC12 cells were transfected with scrambled siRNA or siRNA against *EZH2*. The mRNA levels of *EZH2* and *RET* were assessed by qPCR and normalized against β-actin (mean ± SD, *n* = 3). (**C**) The mRNA levels of *Ret* were assessed in *Kras^G12D^Setd2^–/–^* mouse lung tumor cells or JHRCC12 cells infected with control retrovirus or retrovirus expressing SETD2ΔN by qPCR. Data were normalized against β*-*actin (mean ± SD, *n* = 3). (**D**) Representative ATAC-Seq tracks at the *Ret* locus in *Kras^G12D^* and *Kras^G12D^Setd2^–/–^* mouse lung tumor cells. (**E**) ATAC-Seq tracks at the *Ret* locus in *Kras^G12D^* and *Kras^G12D^Setd2^–/–^* mouse lung tumor cells and ENCODE data showing ChIP-Seq tracks for H3K4me1 and H3K27ac in mouse lung tissues. (**F**) Tumor cells dissociated from *Kras^G12D^* and *Kras^G12D^Setd2^–/–^* mouse lung tumors were assessed by ChIP-qPCR using the indicated antibodies for the promoter and intron 4 of *Ret*. Data shown are the percent input (mean ± SD, *n* = 3). (**G**) JHRCC12 cells infected with control retrovirus or retrovirus expressing SETD2ΔN were assessed by ChIP-qPCR as in **F**. **P < 0.01; ***P < 0.001 by Student’s *t* test.

To determine whether a similar mechanism of ETV1 regulation contributes to induction of PRC2 targets upon SETD2 loss, we focused on RET, a receptor tyrosine kinase, which was upregulated in *Kras^G12D^Setd2^–/–^* mouse lung tumors compared with *Kras^G12D^* tumors (Figure 5A). To confirm that RET is a PRC2 target and is regulated by EZH2, we first determined whether knockdown of EZH2 led to upregulation of RET. Indeed, knockdown of *EZH2* resulted in upregulation of *RET* in JHRCC12 cells (Figure 5B), supporting that RET is a PRC2 target. Retroviral transduction of SETD2ΔN in both mouse *Kras^G12D^Setd2^–/–^* lung cancer cells and JHRCC12 kidney cancer cells downregulated RET (Figure 5C), supporting a regulation of RET by SETD2 in both lung and kidney cancers. The ATAC-Seq tracks at the mouse *Ret* locus revealed increased chromatin accessibility at both promoter and intronic regions in response to SETD2 loss (Figure 5D). Notably, the ATAC-Seq peak at the intron 4 of mouse *Ret* coincides with H3K4me1 ChIP-Seq peaks derived from mouse lung tissues based on ENCODE data (38) (Figure 5E), and the ATAC-Seq peak at the intron 4 likely contains an intronic enhancer. ChIP-qPCR showed that SETD2 loss greatly increased H3K27ac at the intron 4 of *Ret* in dissociated mouse lung tumor cells (Figure 5F), suggesting that SETD2 suppresses this intronic enhancer and thereby downregulates the expression of *Ret*. Consistently, retroviral transduction of SETD2ΔN in JHRCC12 cells reduced H3K27ac at the intron 4 enhancer of *RET* (Figure 5G), supporting a conserved regulation of RET by SETD2 in human ccRCC.

**Figure 6:**
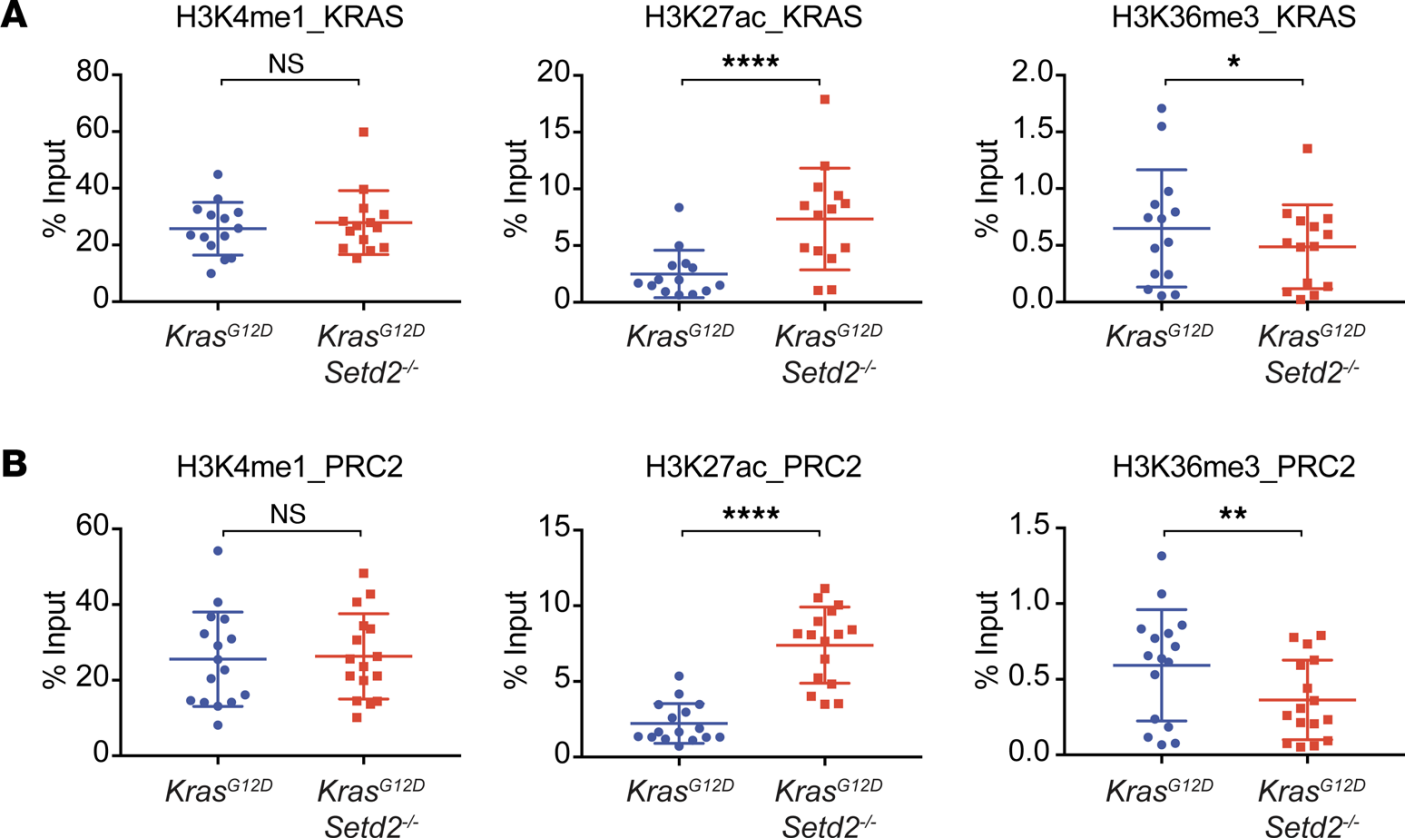
*Setd2* deficiency activates enhancers in the PRC2 and KRAS signature genes. (**A** and **B**) The intron and intergenic regions in the upregulated KRAS (**A**) and PRC2 (**B**) signature genes that display increased chromatin accessibility determined by ATAC-Seq upon *Setd2* deletion in *Kras^G12D^* mouse lung tumors were assessed by ChIP-qPCR using the indicated antibodies. Each data point represents a genomic locus. Data shown are the percent input (mean ± SD, *n* = 14 for KRAS signature genes and *n* = 16 for PRC2 signature genes). *P < 0.05; **P < 0.01; ****P < 0.0001 by Student’s *t* test.

We next investigated whether *Setd2* deletion also activates enhancers of other KRAS and PRC2 signature genes. The intronic and intergenic regions in the upregulated KRAS and PRC2 signature genes that displayed increased chromatin accessibility upon *Setd2* deletion and coincided with H3K4me1 or H3K27ac ChIP-Seq peaks in mouse lung tissue, based on ENCODE data set, were selected for further analyses using ChIP-qPCR. Indeed, *Setd2* deficiency significantly increased H3K27ac levels in these putative enhancers associated with upregulation of these KRAS and PRC2 signature genes (Figure 6). These data support a tumor suppressor model in which SETD2 loss activates enhancers to drive oncogenic transcriptional output through increased chromatin accessibility and enhancer activity to promote lung tumorigenesis.

### SETD2 loss increases histone chaperone recruitment to chromatin and enhances histone exchange.

**Figure 7:**
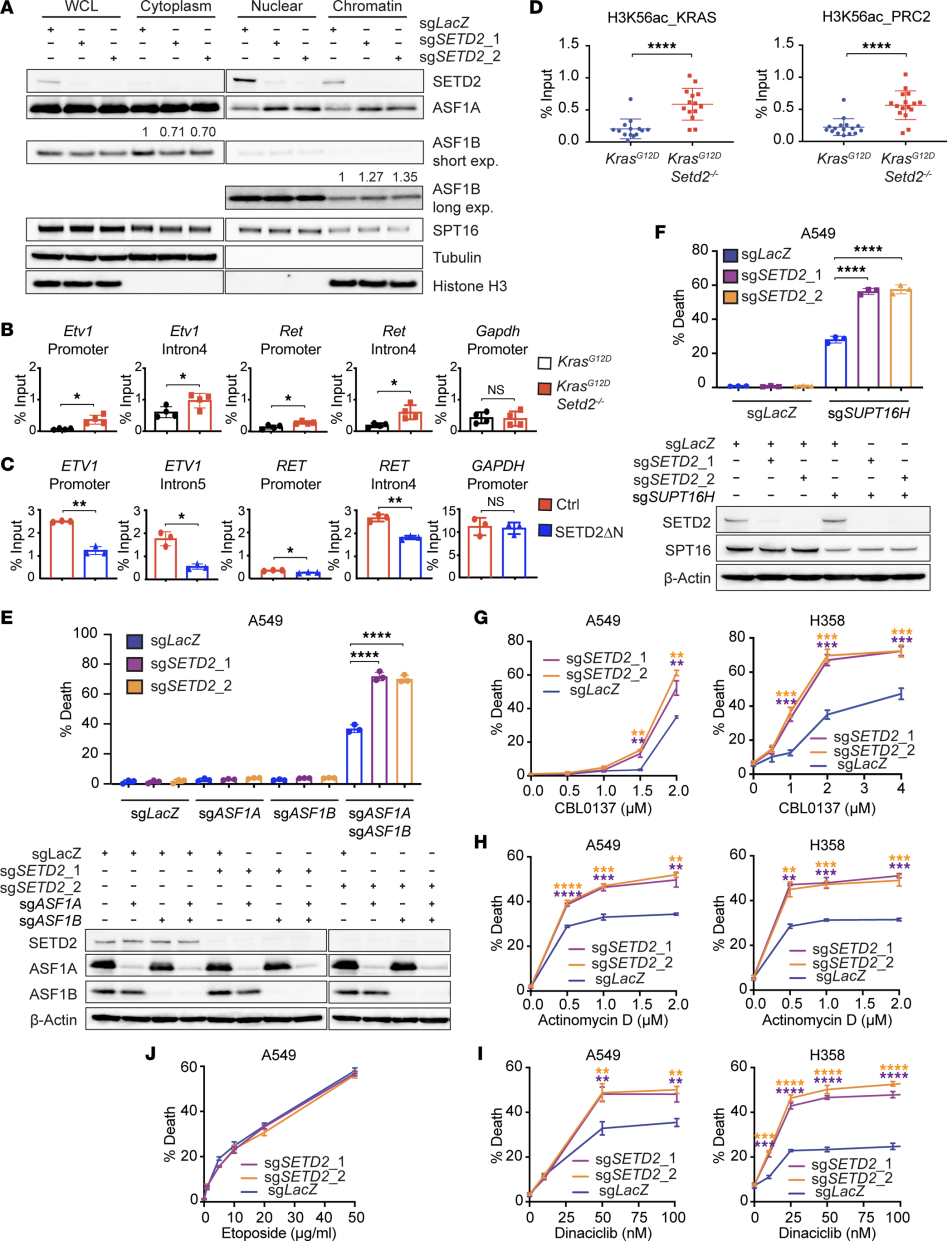
SETD2 loss increases histone chaperone recruitment and sensitizes cancer cells to inhibition of histone chaperones and transcription. (**A**) Whole cell lysates (WCL), cytoplasm, nuclear, and chromatin fractions of A549 cells transduced with the indicated sgRNAs were analyzed by immunoblots. (**B**) Primary cells from *Kras^G12D^* and *Kras^G12D^Setd2^–/–^* mouse lung tumors were assessed by ChIP-qPCR for H3K56ac at the indicated genomic regions. Data shown are the percent input (mean ± SD, *n* = 3). (**C**) JHRCC12 cells ± transduction of SETD2ΔN were assessed by ChIP-qPCR for H3K56ac at the indicated genomic regions. Data shown are the percent input (mean ± SD, *n* = 3). (**D**) The intron and intergenic regions in the upregulated KRAS and PRC2 signature genes that display increased chromatin accessibility upon *Setd2* deletion were assessed by ChIP-qPCR for H3K56ac. Each data point represents a genomic locus. Data shown are the percent input (mean ± SD, *n* = 14 for KRAS signature genes and *n* = 16 for PRC2 signature genes). (**E**) A549 cells transduced with the indicated sgRNAs were subject to FACS analyses following annexin V staining (mean ± SD, *n* = 3) and immunoblot analyses. (**F**) A549 cells transduced with lentivirus expressing the indicated sgRNAs were subject to FACS analyses following annexin V staining (mean ± SD, *n* = 3) and immunoblot analyses. (**G**–**I**) A549 and H358 cells transduced with lentivirus expressing sgRNAs targeting either *LacZ* or *SETD2* were treated with CBL0137 (**G**), actinomycin D (**H**), or dinaciclib (**I**) at the indicated concentrations. Cell death was quantified by annexin V staining (mean ± SD, *n* = 3). (**J**) The indicated A549 cells were treated with etoposide at the indicated concentrations. Cell death was quantified by annexin V staining (mean ± SD, *n* = 3). *P < 0.05; **P < 0.01; ***P < 0.001; ****P < 0.0001 by Student’s *t* test. Purple asterisk, comparing sgLacZ with sgSETD2_1; orange asterisk, comparing sgLacZ with sgSETD2_2.

In yeast, H3K36me3 mediated by Set2 (the ortholog of SETD2) has been shown to suppress the interaction of H3 with the histone chaperones Asf1 and Spt16, and this process in turn reduces the histone exchange over coding regions (6, 39, 40). Accordingly, we hypothesized that a SETD2 loss–induced increase in chromatin accessibility may be caused by aberrant histone chaperone recruitment to chromatin that increases nucleosome disassembly (39, 41). Indeed, lentiviral transduction of Cas9 and sgRNAs targeting *SETD2* in A549 cells increased the chromatin association of ASF1A and ASF1B (human orthologs of Asf1) but not SPT16 (the human ortholog of Spt16) (Figure 7A). Consistent with these findings, we recently reported that retroviral transduction of SETD2ΔN in JHRCC12 cells greatly reduced the chromatin association of ASF1A and ASF1B but not SPT16 (29). Of note, yeast Set2 can catalyze mono-, di-, and tri-methylation of H3K36, whereas mammalian SETD2 is only responsible for the trimethylation of H3K36 (3–6).

A Set2 loss–induced increase in the chromatin recruitment of Asf1 has been shown to result in an enrichment of H3K56ac due to Asf1-mediated exchange of H3K56ac (6, 39). Because K56 is located in the H3 histone-fold domain, acetylation of H3K56 occurs on soluble histones rather than on chromatin and is dependent on ASF1 (42, 43). Consistently, *Setd2* deletion in *Kras^G12D^* mouse lung tumor cells resulted in a significant increase of H3K56ac levels at the promoters and intronic enhancers at the *Etv1* and *Ret* but not *Gapdh* loci (Figure 7B), and this correlated with increased chromatin accessibility determined by ATAC-Seq (Figure 3F and Figure 5D). Retroviral transduction of SETD2ΔN in JHRCC12 cells greatly reduced H3K56ac levels at the *ETV1* and *RET* but not *GAPDH* loci (Figure 7C). Furthermore, H3K56ac levels were significantly increased in the selected putative enhancers of KRAS and PRC2 signature genes that were upregulated upon *Setd2* deletion in mouse *Kras^G12D^* lung tumors (Figure 7D). Collectively, loss of SETD2-mediated H3K36me3 is associated with increased chromatin recruitment of histone chaperones ASF1A/B, enhanced histone exchange, and increased H3K56ac deposition in both kidney and lung cancers, all of which is analogous to the findings observed in *Set2*-deficient yeast (6, 39, 40).

### SETD2 loss sensitizes cancer cells to the inhibition of histone chaperones, FACT complex, or transcriptional elongation.

We hypothesize that SETD2-deficient cancer may be more sensitive to the inactivation of ASF1A and ASF1B than SETD2-proficient cancer if SETD2 loss–induced increase in histone exchange is required for creating a permissive epigenetic landscape to enhance oncogenic transcriptional output. Indeed, lentiviral transduction of 2 independent sgRNAs targeting *SETD2* sensitized A549 cells to undergoing apoptosis in response to inactivation of both *ASF1A* and *ASF1B* through lentiviral transduction of Cas9 and sgRNAs (Figure 7E). Inactivation of either *ASF1A* or *ASF1B* alone failed to induce apoptosis, suggesting a potential genetic redundancy. Moreover, SETD2 loss sensitized A549 to apoptosis triggered by inactivation of *SUPT16H* (gene name of SPT16) (Figure 7F). Although loss of H3K36me3 had a minimal impact on the chromatin association of SPT16, H3K36me3 loss–induced chromatin recruitment of ASF1A/B potentially leads to increased chromatin accessibility to other histone chaperones, including SPT16, establishing a feed-forward amplification loop for the maintenance of open chromatin status. Of note, SPT16 is a component of the facilitates chromatin transcription (FACT) complex that facilitates transcription elongation. Hence, inhibition of SPT16 or the FACT complex may affect both chromatin dynamics and transcription. Similar to inactivation of *SUPT16H*, SETD2 loss of function sensitized A549 and H358 (a human *KRAS* mutant lung cancer cell line) to apoptosis triggered by the FACT complex inhibitor CBL0137 (44) (Figure 7G). Notably, restoration of H3K36me3 in JHRCC12 through retroviral transduction of SETD2ΔN suppressed apoptosis triggered by inactivation of *ASF1A/B* or *SUPT16H* as well as CBL0137 treatment (29), supporting a critical role of histone chaperons in the tumor suppressor function of SETD2 across different cancer types.

**Figure 8:**
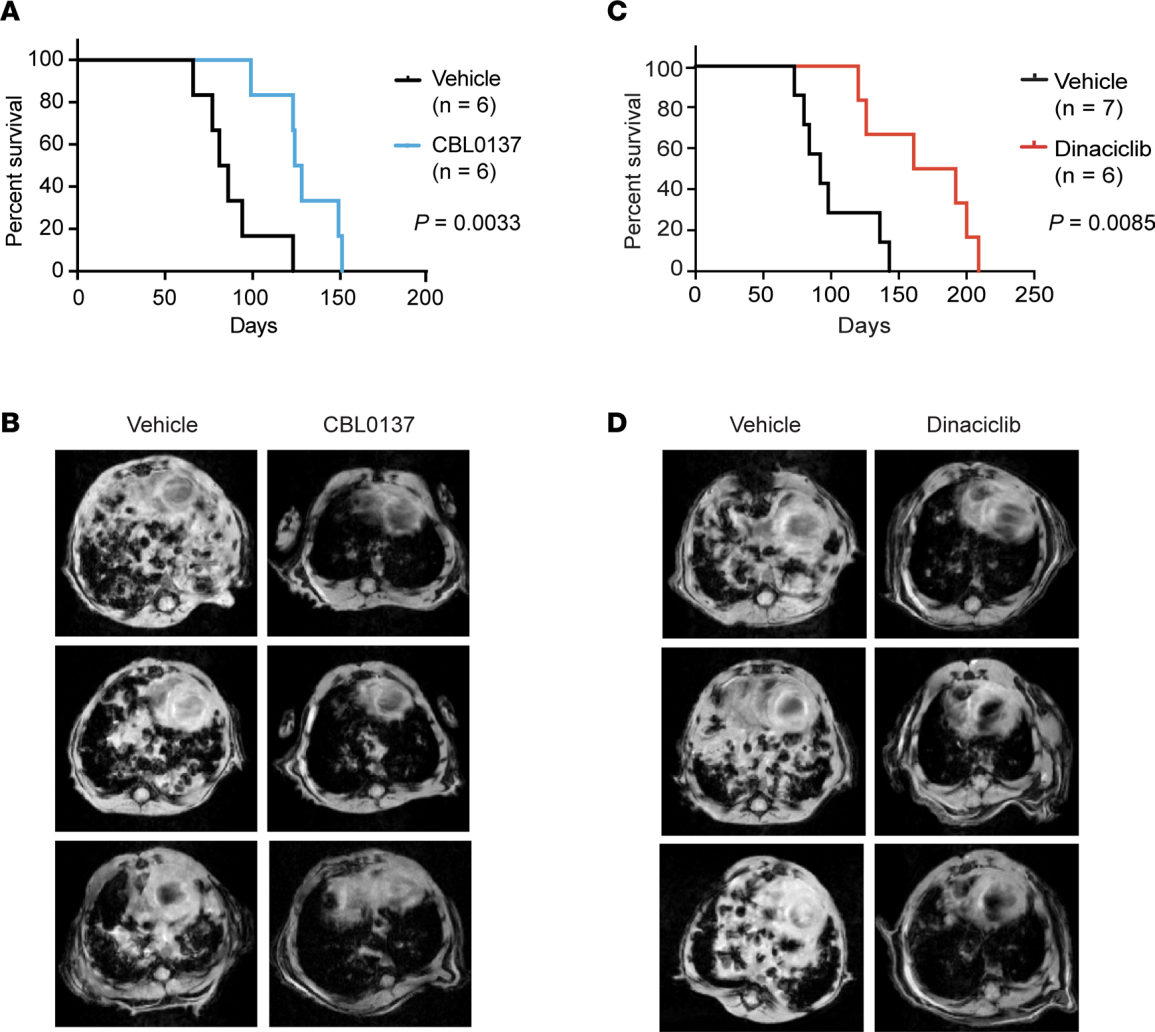
The FACT complex and CDK9 inhibitors suppress the growth of *Kras^G12D^Setd2^–/–^* lung tumors in vivo. (**A**) Kaplan-Meier survival curves of *Kras^LSL–G12D/+^ Setd2^fl/fl^* mice treated with vehicle (*n* = 6) or CBL0137 (*n* = 6, 60 mg/kg, twice weekly) starting at 4 weeks after adeno-Cre infection. CBL0137 versus vehicle, *P* = 0.0033 (Mantel-Cox test). (**B**) *Kras^LSL–G12D/+^ Setd2^fl/fl^* mice infected with adeno-Cre were treated with vehicle or CBL0137 for 4 weeks starting at 4 weeks after adeno-Cre infection. Representative MRI images of lungs were obtained 3 weeks later. (**C**) Kaplan-Meier survival curves of *Kras^LSL–G12D/+^ Setd2^fl/fl^* mice treated with vehicle or dinaciclib (20 mg/kg, 3 times/week) starting at 4 weeks after adeno-Cre infection. Dinaciclib versus vehicle, *P* = 0.0085 (Mantel-Cox test). (**D**) Representative MRI images of lungs of *Kras^LSL–G12D/+^ Setd2^fl/fl^* mice infected with adeno-Cre and treated with vehicle or dinaciclib for 7 weeks starting at 4 weeks after adeno-Cre infection.

Given that *Setd2* deficiency activates oncogenic transcriptional output to promote tumorigenesis, we reason that SETD2-deficient cancer may be more sensitive to inhibitors of transcription. Indeed, lentiviral transduction of Cas9 and sgRNAs targeting *SETD2* sensitized A549 and H358 to apoptosis triggered by the commonly used RNA transcription inhibitor, actinomycin D (Figure 7H). Retroviral transduction of SETD2ΔN reduced actinomycin D–induced apoptosis in *SETD2* mutated JHRCC12 cells (Supplemental Figure 4A). We next tested dinaciclib, a targeted therapeutic agent entering clinical trials that inhibits CDK9 and transcriptional elongation (45, 46). CDK9 is a serine-threonine kinase that forms the catalytic core of the p-TEFb complex and, in the presence of cyclin T, phosphorylates Ser2 in the C-terminal domain (CTD) of Pol II to stimulate transcription elongation (47). In A549, H358, and JHRCC12 cells, SETD2 loss of function sensitized cells to apoptosis triggered by dinaciclib (Figure 7I and Supplemental Figure 4B). It is noteworthy that SETD2 loss did not sensitize A549 cells to the topoisomerase II inhibitor etoposide (Figure 7J), indicating that SETD2 loss does not simply lower apoptotic threshold. Lastly, we assessed the in vivo therapeutic effect of CBL0137 and dinaciclib in mice bearing *Kras^G12D^Setd2^–/–^* lung tumors. Both CBL0137 and dinaciclib treatment markedly suppressed the growth of *Kras^G12D^Setd2^–/–^* lung tumors and significantly prolonged the survival of mice (Figure 8). In summary, our mechanistic elucidation of the tumor suppressor function of SETD2 has identified therapeutic vulnerabilities and actionable therapeutic targets for the treatment of SETD2-deficient cancers.

## Discussion

**Figure 9:**
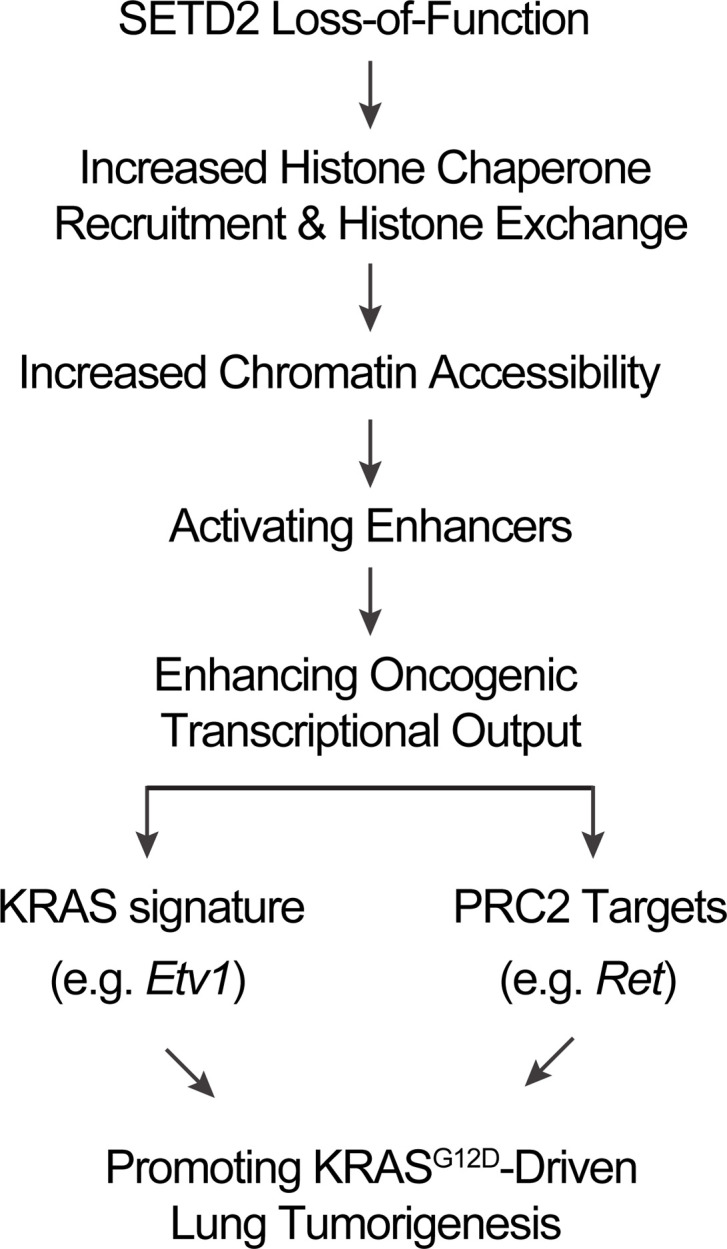
A schematic summarizing the tumor suppressor mechanisms of SETD2 in KRAS^G12D^-driven lung cancer.

Here, we employed a *Setd2*–conditional KO mouse model to elucidate the molecular mechanisms by which *Setd2* deficiency cooperates with KRAS to promote lung tumorigenesis. Homozygous deletion of *Setd2* significantly accelerated both the initiation and progression of Kras^G12D^-driven lung tumors with reduced mouse survival. Our integrated epigenetic and transcriptomic analysis revealed that *Setd2* deficiency resulted in a coordinated reprogramming of the epigenome and the transcriptome, and this reprogramming in turn amplifies the oncogenic signatures to promote *Kras^G12D^*-driven lung tumorigenesis (Figure 9). We have identified KRAS and PRC2 as main oncogenic pathways activated by *Setd2* deficiency in *Kras^G12D^Setd2^–/–^* mouse lung tumors. Using *SETD2*-mutant metastatic cell line and xenograft models derived from patients with ccRCC, we recently reported that H3K36me3 restoration greatly reduced distant metastases of ccRCC in mice (29). Notably, our integrated analyses of RNA-Seq, ATAC-Seq, and ChIP-Seq data uncovered an epigenetic tumor suppressor model of SETD2 common to both kidney and lung cancers in which SETD2 loss creates a permissive epigenetic landscape for the cooperating oncogenic drivers to further amplify transcriptional output. This helps explain why *SETD2* mutations occur in a wide variety of human cancers and are associated with diverse oncogenic drivers.

Because SETD2-dependent H3K36me3 is enriched in actively transcribed gene bodies, it has been considered as a histone mark of active transcription. However, our results in both lung and kidney cancers show that loss of SETD2 counterintuitively increases oncogenic transcriptional output. This paradox was resolved by our findings that SETD2-mediated cotranscriptional deposition of H3K36me3 in gene bodies indeed maintains the closed chromatin structure by suppressing the binding of histone chaperones and reducing histone exchange (Figure 9). This is analogous to the reported role of Set2-mediated H3K36me3 in preventing cryptic transcription initiation in yeast through inhibition of histone exchange (6). Overall, our data support a model in which loss of SETD2-mediated H3K36me3 enhances histone exchange through the recruitment of ASF1A/B to chromatin, resulting in increased chromatin accessibility to other histone chaperones such as SPT16, transcription factors, or chromatin remodeling complexes to establish a feed-forward amplification loop (Figure 9). Consequently, loss of SETD2 creates an epigenetic landscape consisting of widespread open chromatin that amplifies oncogenic transcriptional output through aberrant activation of enhancers.

Here, our approaches integrating a genetically engineered mouse model, RNA-Seq, and ATAC-Seq have established an epigenetic tumor suppressor model of SETD2 that lays the foundation for developing mechanism-based therapeutic strategies for SETD2-deficient cancers. Of note, mutations in *SETD2* have been reported to confer resistance to DNA-damaging chemotherapy in leukemia (48). Consistent with the requirement of histone chaperones for the establishment of an epigenetic landscape permissive for tumorigenesis upon SETD2 loss, SETD2-deficient cancer cells were more sensitive to genetic and chemical inhibition of histone chaperones. We also found that SETD2-deficient cancer cells were more sensitive to transcriptional inhibitors, including the CDK9 inhibitor dinaciclib, which is in accordance with the notion that SETD2 loss–induced upregulation of oncogenic transcriptional programs is required for tumor maintenance. Importantly, we have demonstrated the in vivo therapeutic efficacy of the FACT complex inhibitor CBL0137 and dinaciclib in mice bearing *Kras^G12D^Setd2^–/–^* lung tumors. These mechanism-based therapeutic strategies will likely provide an avenue for the treatment of *SETD2* mutant lung cancer and other cancers.

## Methods

### Mice.

*Kras^LSL–G12D/+^* transgenic mice were obtained from the Jackson Laboratory. The *Setd2^fl/fl^* mice were generated by Beijing Biocytogen Co. Ltd. *Kras^LSL–G12D/+^* and *Setd2^fl/fl^* mice were bred to generate *Kras^LSL–G12D/+^ Setd2^fl/+^* and *Kras^LSL–G12D/+^ Setd2^fl/fl^* mice. All animals were maintained on a mixed C57BL/6J × 129SvJ genetic background. Intranasal instillation of 2.5 × 10^7^ plaque-forming units (pfu) of adenovirus-expressing Cre (Viral Vector Core Facility, University of Iowa, Iowa City, Iowa, USA) was performed on mice at 6–10 weeks of age as previously described (49). Tumor growth was monitored by MRI scans. Sex-matched *Kras^LSL–G12D/+^ Setd2^fl/fl^* mice, 4 weeks after adeno-Cre infection, were randomized into vehicle control and dinaciclib or CBL0137 treatment groups. CBL0137 (Selleck Chemicals) was formulated in 50 mg/mL Captisol and administered i.v. twice weekly at 60 mg/kg for 4 weeks. Dinaciclib (Selleck Chemicals) was formulated in 20% hydroxypropyl β-cyclodextrin (MilliporeSigma) and administered i.p. 3 times weekly at 20 mg/kg. Lung tumor growth was assessed by MRI scans at 7 weeks after the first treatment. The body weights of the mice were monitored twice weekly.

### IHC and immunofluorescence.

Lungs of mice were perfused with 10% buffered formalin via the trachea and fixed in 10% formalin overnight at room temperature. The fixed lungs were processed and embedded in paraffin. Total lung and tumor areas were measured from H&E-stained slides using ImageJ software (NIH). Tumor burden was expressed as the percentage of lung occupied by tumor ([area tumor/area lung] ***×*** 100). IHC for phospho-H3S10 and TUNEL assays were performed by the Molecular Cytology Core Facility at the MSKCC. IHC for Ki67, cleaved caspase-3, phospho-ERK, H3K36me3, H3K4me3, H3K4me1, and H3K27ac were conducted by the Laboratory of Comparative Pathology at MSKCC. Quantification of Ki67, phospho-H3S10, H3K4me3, H3K4me1, H3K27ac, cleaved caspase-3, and TUNEL staining was performed using ImageJ software. The following primary antibodies were used for IHC: phospho-ERK1/2 (Cell Signaling Technology, 4370, 1:1,000 dilution), Ki67 (Abcam, ab16667, 1:100 dilution), cleaved caspase-3 (Cell Signaling Technology, 9661, 1:250 dilution), H3K36me3 (Abcam, ab9050, 1:1,000 dilution), H3K4me1 (Abcam, ab8895, 1:10,000 dilution), H3K4me3 (Abcam, ab8580, 1:1,500 dilution), and H3K27ac (Abcam, ab4729, 1:750 dilution). For immunofluorescence, H3K36me3 (Abcam, ab9050, 1:1,000 dilution) and H3K27me3 (MilliporeSigma, 07-449, 1:1,000 dilution) were used as primary antibodies; goat anti–rabbit IgG (H+L) Alexa Fluor 568 (Thermo Fisher Scientific, A11036, 1:2,000 dilution) was used as a secondary antibody. All histopathological analyses were assisted by a board-certified pathologist.

### Isolation and transient culture of mouse lung tumor cells.

Distinct lung tumors were dissected from mice. Tumors were minced and digested in advanced DMEM/F12 containing liberase for 1 hour at 37°C. Dissociated tumor samples were filtered through 40 μm strainers and washed with cold HBSS twice. Cells were cultured in advanced DMEM/F12 supplemented with penicillin/streptomycin (Thermo Fisher Scientific), NEAA (Thermo Fisher Scientific), GlutaMAX (Thermo Fisher Scientific), 10 mM HEPES (Thermo Fisher Scientific), B-27 (Thermo Fisher Scientific), 50 ng/mL EGF, 8 ng/mL huFGF (Thermo Fisher Scientific), 3 ng/mL HGF (Thermo Fisher Scientific), 10 mM nicotinamide (MilliporeSigma), 1.25 mM N-Acetylcysteine (MilliporeSigma), and 0.25 μg/mL amphotericin B (Thermo Fisher Scientific).

### Cell culture and viability assay.

A549, H358, and 786-O cell lines were obtained from the American Type Culture Collection (ATCC) and cultured according to the recommendations of ATCC. JHRCC12 cell line was generated and cultured as previously described (37). Cell death was quantified by annexin V (BioVison) staining, followed by flow cytometric analyses using an LSRFortessa (BD Biosciences) as described (50). Data were analyzed using FACSDiva (BD Biosciences). The following chemicals were used in the viability assays: actinomycin D (MilliporeSigma), dinaciclib (Selleck Chemicals), CBL0137 (Cayman Chemical), and etoposide (MilliporeSigma). Viability of H358 cells treated with CBL0137, actinomycin D, or dinaciclib was assessed at 24 hours after treatment. Viability of A549 treated with CBL0137, actinomycin D, and dinaciclib was assessed at 36 hours, 48 hours, and 72 hours after treatment, respectively.

### Soft agar colony formation assays.

Cells (2 × 10^5^) were added to 4 mL of growth media plus 0.3% Noble Agar (Difco) and layered onto a 4 mL bed of growth media plus 0.6% Noble Agar in a 6 cm tissue culture dish. Cells were fed every 3 days with 1.5 mL of growth media. The colonies with diameters larger than 100 μm were quantified at 4 weeks using GelCount (Oxford Optronix).

### Plasmid construction, RNA interference, and CRISPR/Cas9-mediated genome editing.

Human *SETD2* with deletion of the N-terminal 3,723 bp was tagged with 3xFLAG at the N-terminus and cloned into pBABE-puro (Addgene). For CRISPR/Cas9-mediated KO and deletion, sgRNAs were designed using Optimized CRISPR Design and cloned into lentiCRISPRv2 (51). All constructs were confirmed by DNA-Seq. Lentivirus was produced by cotransfection of 293T cells with pCMVDR8.2 and pHCMV.VSVG using Lipofectamine 2000 (Thermo Fisher Scientific) as described (50). The siRNA oligonucleotides against *EZH2* (Thermo Fisher Scientific, 4392420) and scrambled siRNA oligonucleotides (Thermo Fisher Scientific, 4390847) were reverse transfected using Lipofectamine RNAiMAX (Thermo Fisher Scientific) as described (52). The sequences of siRNAs and sgRNAs were summarized in Supplemental Table 2.

### Reverse transcription and qPCR.

Total RNA was extracted from cells or tissues using Trizol (Thermo Fisher Scientific) according to the manufacturer’s instructions. Reverse transcription was performed with oligo-dT plus random decamer primers (Thermo Fisher Scientific) using Superscript II (Thermo Fisher Scientific). qPCR was performed with SYBR green master mix (Thermo Fisher Scientific) in duplicates using the indicated gene-specific primers on a ViiA 7 Real-Time PCR System (Thermo Fisher Scientific). Data were analyzed as described previously by normalization against β-actin (50). Primers for qPCR are listed in Supplemental Table 2.

### Histone extraction and immunoblot analysis.

To extract histones, cells were lysed in lysis buffer (0.5% Triton X-100, 150 mM NaCl, 1.5 mM MgCl_2_, 4 mM sodium butyrate, 100 mM Tris [pH 7.5]; MilliporeSigma) supplemented with complete protease inhibitors (Roche) for 10 minutes on ice, washed once with the lysis buffer, and reresuspended in 0.4N HCl for 1 hour on ice. After centrifugation at 15,000*g* at 4°C for 15 minutes, proteins in the supernatant were precipitated with 10× volume of acetone at –20°C overnight. The pellet was then washed once with cold acetone and resuspended in deionized water. To prepare whole cell lysates, cultured cells were lysed in RIPA buffer; dissected tumors were minced to pieces in RIPA buffer and homogenized by FastPrep-24 homogenizer (MP Biomedicals). Protein concentration was determined by BCA kit (Pierce). Extracted histones or whole cell lysates were resolved by 10 % or 4%–12% NuPAGE gels (Thermo Fisher Scientific) and transferred onto PVDF membranes (Immobilon-P, MilliporeSigma). Antibody detection was accomplished using enhanced chemiluminescence method (Western Lightning, PerkinElmer) and LAS-3000 Imaging system (FUJIFILM). Antibodies used for immunoblot analyses are listed as follows: anti-ETV1 (Abcam, ab81086, 1:500 dilution), anti-SETD2 (MilliporeSigma, HPA042451, 1:500 dilution), anti-SPT16 (Cell Signaling Technology, 12191, 1:1,000 dilution), anti–β-actin (MilliporeSigma, A1978, 1:10,000 dilution), anti-H3K4me3 (Abcam, ab8580, 1:1,000 dilution), anti-H3K27me3 (Cell Signaling Technology, 9733, 1:1,000 dilution), anti-H3K27ac (Abcam, ab4729, 1:1,000 dilution), anti-H3K36me2 (MilliporeSigma, 07-369, 1:1,000 dilution), anti-H3K36me3 (Abcam, ab9050, 1:1,000 dilution), anti-H3 (Cell Signaling Technology, 14269, 1:1,000 dilution), anti-ASF1A (Cell Signaling Technology, 2990S, 1:1,000 dilution), and anti-ASF1B (Cell Signaling Technology, 2902S, 1:1,000 dilution). Immunoblots were quantified using ImageJ (NIH) software.

### ChIP-qPCR.

Cells (2 × 10^6^) were cross-linked with 1% paraformaldehyde for 10 minutes at room temperature and quenched by glycine. Cells were washed with cold PBS, centrifuged at 360*g* at 4°C for 5 minutes, and lysed. After sonication, samples were spun down and incubated with 1 μg primary antibody for each ChIP experiment at 4°C overnight. Magnetic beads (Thermo Fisher Scientific) were added the next day and incubated at 4°C for 2 hours. Samples were then washed, and histone complexes were eluted. The eluted samples were treated with RNase A and proteinase K, reversed crosslink, and purified with Qiagen PCR purification kit. The purified DNA samples were subjected to qPCR using the indicated gene-specific primers listed in Supplemental Table 2. Antibodies used for ChIP experiments are listed as follows: H3K4me1 (Abcam, ab8895), H3K4me3 (Active Motif, 39159), H3K27me3 (Cell Signaling Technology, 9733), H3K27ac (Abcam, ab4729), H3K36me3 (Abcam, ab9050), H3K56ac (Millipore, 07-677), and rabbit IgG (Abcam, ab171870). Data were normalized as percentage of input.

### Dual-luciferase reporter assay.

The indicated intron 4 sequence of mouse *Etv1* and intron 5 sequence of human *ETV1* were amplified from genomic DNA by PCR and cloned into pGL2-Promoter (pGL2-pro) vector (Promega) upstream of the SV40 promoter. A549 cells were cotransfected with pGL2-pro or pGL2-pro containing the DNA fragment from the intron 4 of mouse *Etv1* or the intron 5 of human *ETV1* together with the pRL-SV40 plasmid (Promega) using Lipofectamine 2000 (Thermo Fisher Scientific). The firefly and *Renilla* luciferase activities were assessed 36 hours after transfection using the Dual-Luciferase Reporter Assay System (Promega). The firefly luciferase activity was normalized against the *Renilla* luciferase activity.

### ATAC-Seq and analysis.

In total, 50,000 dissociated mouse lung tumor cells were used for the transposition reaction at 37°C for 30 minutes. After purification of the DNA with the MinElute PCR purification kit (Qiagen), material was amplified for 5 cycles as described previously (28). Additional PCR cycles were evaluated by real-time PCR. Final product was cleaned by AMPure Beads at a 1.5***×*** ratio. Libraries were sequenced by the Integrated Genomics Operation Core Facility at MSKCC on a HiSeq 2500 1T in a 50 bp/50 bp paired end run, using the TruSeq SBS Kit v3 (Illumina). On average, 50 million paired reads were generated per sample. Raw reads were trimmed and filtered for quality using Trimmomatic (53). Trimmed reads were mapped to the mm10 genome assembly using Bowtie2 (54), and nonuniquely mapping reads were removed. The reads were adjusted by shifting all positive-strand reads 4 bp downstream and all negative-strand reads 5 bp upstream to center the reads on the transposase binding event. Peak calling was performed on each replicate, and all replicates were merged together using MACS2 with “--extsize 200 --shift -100 --nomodel” parameters (55). Using MACS2 bdgcmp with “-m ppois” parameter, the Poisson *P* value was generated for each individual replicate. To find a set of peaks that is reproducible across replicates, we calculated the irreproducible discovery rate (IDR) (56) on peaks called from merged samples but scored with *P* values separately in each replicate of each cell type. We excluded peaks with an IDR greater than 0.05 across every pair of replicates within each cell type. Peaks found reproducibly in each condition were combined to create a genome-wide atlas of accessible chromatin sites. The annotation of the atlas and differential accessibility analysis of the peaks was performed as previously described (57).

Using the MEME-curated CisBP TFBM reference, we scanned the mouse ATAC-Seq peak atlas with FIMO (58) to find peaks likely to contain each TFBM (*P* < 1 ***×*** 10^–5^). Relative transcription factor accessibility was determined using two 1-sided Wilcoxon rank-sign tests comparing the distributions of peak heights for peaks containing FIMO-predicted transcription factor binding sites. GO and KEGG pathway analyses of ATAC-Seq peaks gained in response to *Setd2* deletion in mouse lung tumors (FDR < 0.05) were performed using HOMER (59). ChIP-Seq data of embryonic and postnatal mouse lung tissues were obtained from ENCODE (GSE82758, GSE82997, GSE82980, GSE83004, GSE82654, and GSE82462) (38). The ATAC-Seq peaks gained in intron regions (FDR < 0.05) in differentially expressed genes detected by RNA-Seq (FDR < 0.05) in response to *Setd2* deletion in mouse lung tumors were compared with the ChIP-Seq peaks for H3K4me1 and H3K27ac in mouse lung tissues to assess the presence of intronic enhancers.

### ATAC-Seq peak atlas summary.

A total of 77,025 reproducible ATAC-Seq peaks was identified in mouse lung tumor samples. Among these peaks, 37.5% were found at introns, 34.2% at intergenic regions, 26.7% at promoters, and 1.6% at exons.

### RNA-Seq and analysis.

Distinct *Kras^G12D^* and *Kras^G12D^Setd2^–/–^* mouse lung tumors were dissected and minced into pieces in Trizol (Thermo Fisher Scientific). The minced tumor tissues were then put in Lysing Matrix D tubes (MP Biomedicals) in Trizol and homogenized by FastPrep-24 homogenizer (MP Biomedicals). Total RNA was extracted and cleaned up using RNeasy Mini Kit (Qiagen). Library preparation and sequencing were performed by the Integrated Genomics Operation Core Facility at MSKCC. After RiboGreen quantification and quality control of Agilent BioAnalyzer, 6–15 ng of total RNA underwent amplification (12 cycles) using the SMART-Seq V4 (Clontech) ultra low–input RNA kit for sequencing. In total, 10 ng of amplified cDNA was used to prepare Illumina HiSeq libraries with the Kapa DNA library preparation chemistry (Kapa Biosystems) using 8 cycles of PCR. Samples were barcoded and run on a HiSeq 4,000 in a 50 bp/50 bp paired end run, using the TruSeq SBS Kit v3 (Illumina). On average, 60 million paired reads were generated per sample, and the percentage of mRNA bases was 73% on average. Raw reads were trimmed and filtered for quality using Trimmomatic (53). Processed reads were then aligned against the mm10 version of the mouse genome using STAR (60). For each RefSeq annotated gene, reads overlapping with exon regions were counted using HTSeq (61). Gene-level differential expression analysis was conducted using DESeq2 (62).

Differentially expressed genes detected by RNA-Seq (FDR < 0.05) were subjected to GSEA using the JAVA GSEA 3.0 program (63). The gene sets from the Molecular Signature Database (MSigDB) — including c2 (curated gene sets), c5 (GO gene sets), and c6 (oncogenic signatures gene sets) — were used for the analysis. The composite PRC2 signature was generated by merging the published PRC2 modules in liver cancer (20), MPNST (21), hESCs (25), hematopoietic stem cells (26), and neural progenitor cells (27). The KRAS signature was generated by merging the gene sets from MSigDB, including KRAS.600_UP.V1_UP, KRAS.600.LUNG.BREAST_UP.V1_UP, KRAS.BREAST_UP.V1_UP, KRAS.LUNG_UP.V1_UP, and KRAS.KIDNEY_UP.V1_UP. The PTEN_DN_UP signature was generated by merging PTEN_DN.V1_UP and PTEN_DN.V2_UP data sets from MSigDB.

### Principal component analysis.

Principal component analysis (PCA) plots were generated using normalized RNA-Seq read count data after variance stabilizing transformation in DESeq2 package (62).

### Diamond plots.

Genes in the KRAS or PRC2 signature with both differential expression detected by RNA-Seq (FDR < 0.05) and differentially accessible ATAC-Seq peaks (FDR < 0.05) in response to SETD2 loss were used to generate the diamond plots. Top 20 genes in the KRAS signature and top 25 genes in the PRC2 signature with the highest and lowest fold change (FC) in gene expression were presented. In these plots, the accessibility landscape of each gene is represented by a stack of diamonds corresponding to accessible chromatin sites assigned to the gene. The *y* coordinate of the bottom-most peak in this stack gives the log_2_FC in expression of the gene. The diamonds are colored according to the accessibility change of the ATAC-Seq peak, with blue indicating closing and red indicating opening. The color scale was based on the rank-order of the peak accessibility changes. In Supplemental Figure 2F, the color scale ranges from a log_2_FC of –2.01 to 3.29 for the KRAS signature and of –1.72 to 3.57 for the PRC2 signature.

### Transcriptome analysis of human lung adenocarcinoma.

RNA-Seq data of human lung adenocarcinoma samples were obtained from TCGA. Gene-level differential expression analysis was conducted using DESeq2 to compare transcriptome of *SETD2^MT^* (*n* = 20; 5 of 20 with *KRAS* mutations) with *SETD2*^WT^ (*n* = 210) lung adenocarcinoma samples.

### Data availability.

Raw ATAC-Seq and RNA-Seq data have been deposited in the SRA database under PRJNA885032.

### Statistics.

IHC quantification, tumor burden quantification, qPCR, soft agar colony formation assays, dual-luciferase reporter assays, and cell death assays were analyzed for statistical significance using unpaired 2-tailed Student’s *t* tests (Prism 6.0, GraphPad Software). ChIP-qPCR was analyzed for statistical significance using paired 2-tailed Student’s *t* tests (Prism 6.0, GraphPad Software). Data were presented as mean ± SD, with *P* < 0.05 considered statistically significant unless otherwise stated. The mouse survival curve was determined by the Kaplan-Meier method, and statistical significance was determined by the Mantel-Cox test.

### Study approval.

Animal experiments were approved by the IACUC at MSKCC.

## Author contributions

YX designed and conducted experiments and analyzed data. EHC designed research, analyzed data, and supervised the project. TW, AIY, WZ, and SH conducted some experiments. AMN, YL, and SY analyzed some data. JJH supervised some experiments. MS performed computational analyses. CSL supervised the computational analyses.

## Supplementary Material

Supplemental data

Supplemental table 2

## Version 1 02/22/2023

Electronic publication
