# Supplementary material for: SETD2 regulates chromatin accessibility and transcription to suppress lung tumorigenesis
Source: JCI Insight. 2023 Feb 22;8(4):e154120. doi: 10.1172/jci.insight.154120 (PMC9977508; doi:10.1172/jci.insight.154120)
Supplement: Supplemental data [file jciinsight-8-154120-s178.pdf]

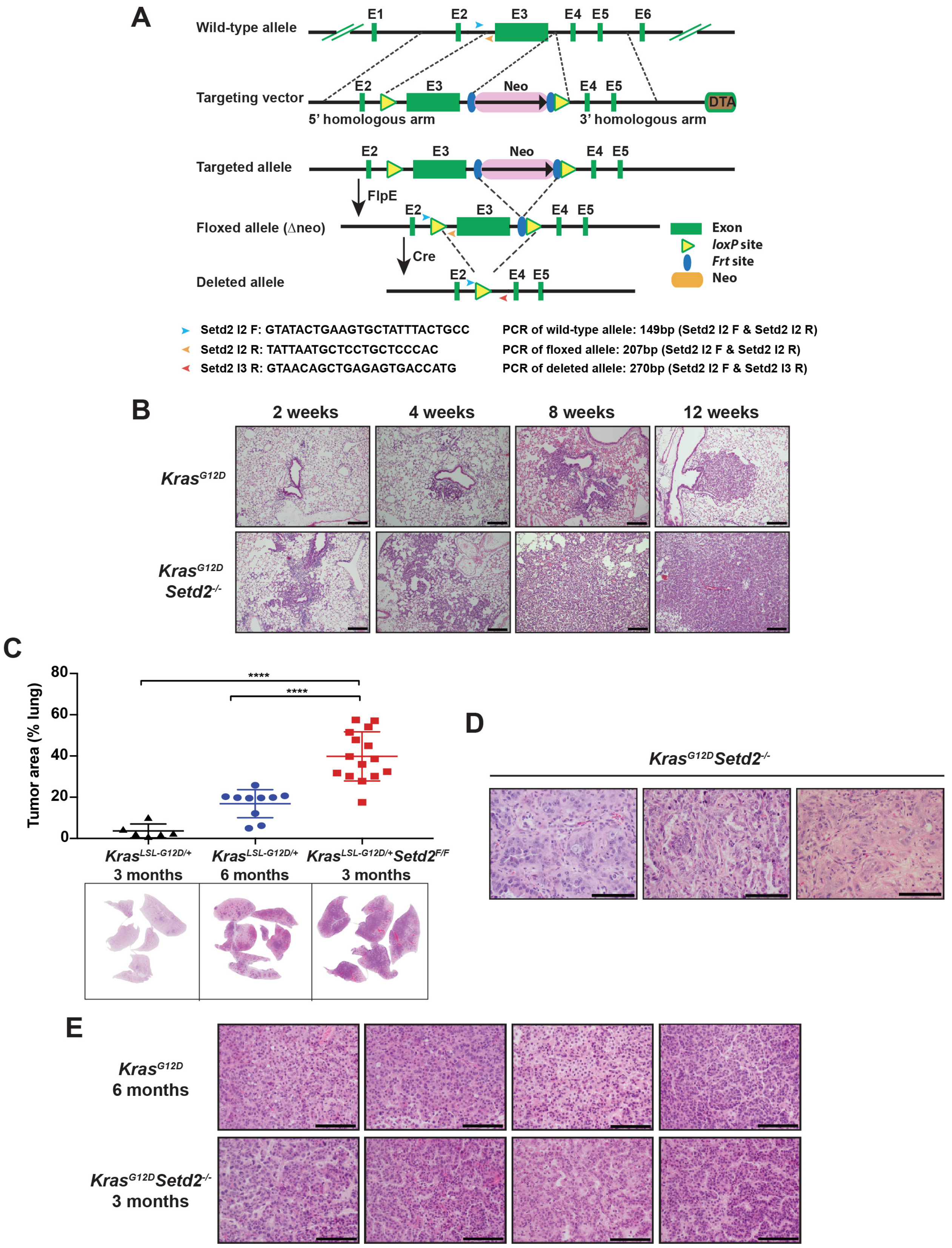
A
Wild-type allele
E1
E2
E3
E4
E5
E6
Targeting vector
E2
E3
Neo
E4
E5
DTA
5' homologous arm
3' homologous arm
Targeted allele
E2
E3
Neo
E4
E5
FlpE
Floxed allele (Δneo)
E2
E3
E4
E5
Cre
Deleted allele
E2
E4
E5
Exon
loxP site
Frt site
Neo
Setd2 I2 F: GTATACTGAAGTGCTATTTACTGCC
Setd2 I2 R: TATTAATGCTCCTGCTCCCAC
Setd2 I3 R: GTAACAGCTGAGAGTGACCATG
PCR of wild-type allele: 149bp (Setd2 I2 F & Setd2 I2 R)
PCR of floxed allele: 207bp (Setd2 I2 F & Setd2 I2 R)
PCR of deleted allele: 270bp (Setd2 I2 F & Setd2 I3 R)
B
2 weeks
4 weeks
8 weeks
12 weeks
Kras G12D
Kras G12D Setd2 -/-
C
Tumor area (% lung)
80
60
40
20
0
****
****
Kras LSL-G12D/+ 3 months
Kras LSL-G12D/+ 6 months
Kras LSL-G12D/+ Setd2 F/F 3 months
D
Kras G12D Setd2 -/-
E
Kras G12D 6 months
Kras G12D Setd2 -/- 3 months

Supplemental Figure 1. (**A**) A schematic diagram of the strategy used to generate *Setd2* conditional knockout mice. (**B**) Representative H&E staining of lungs from $Kras^{LSL\text{-}G12D/+}$ and $Kras^{LSL\text{-}G12D/+}Setd2^{F/F}$ mice at the indicated times after adeno-Cre infection. Scale bars, 200 µm. (**C**) Quantification of lung tumor burden in $Kras^{LSL\text{-}G12D/+}$ and $Kras^{LSL\text{-}G12D/+}Setd2^{F/F}$ mice at the indicated times after adeno-Cre infection (n = 6 for $Kras^{LSL\text{-}G12D/+}$ mice at 3 months after adeno-Cre infection, n = 10 for $Kras^{LSL\text{-}G12D/+}$ mice at 6 months after adeno-Cre infection, n = 15 for $Kras^{LSL\text{-}G12D/+}Setd2^{F/F}$ mice at 3 months after adeno-Cre infection). Percentage tumor area in lungs was calculated. Data are mean ± SD. ****, $P < 0.0001$ (Student's *t*-test). Representative low magnification images of H&E staining of lungs from the indicated mice are shown. (**D**) Representative histopathological images of $Kras^{LSL\text{-}G12D}Setd2^{-/-}$ lung tumors showing focal invasion and juxtatumoral desmoplastic stromal reaction. Scale bars, 100 µm. (**E**) Histopathological images of $Kras^{G12D}$ and $Kras^{G12D}Setd2^{-/-}$ lung tumors prepared for RNA-seq. Scale bars, 100 µm.

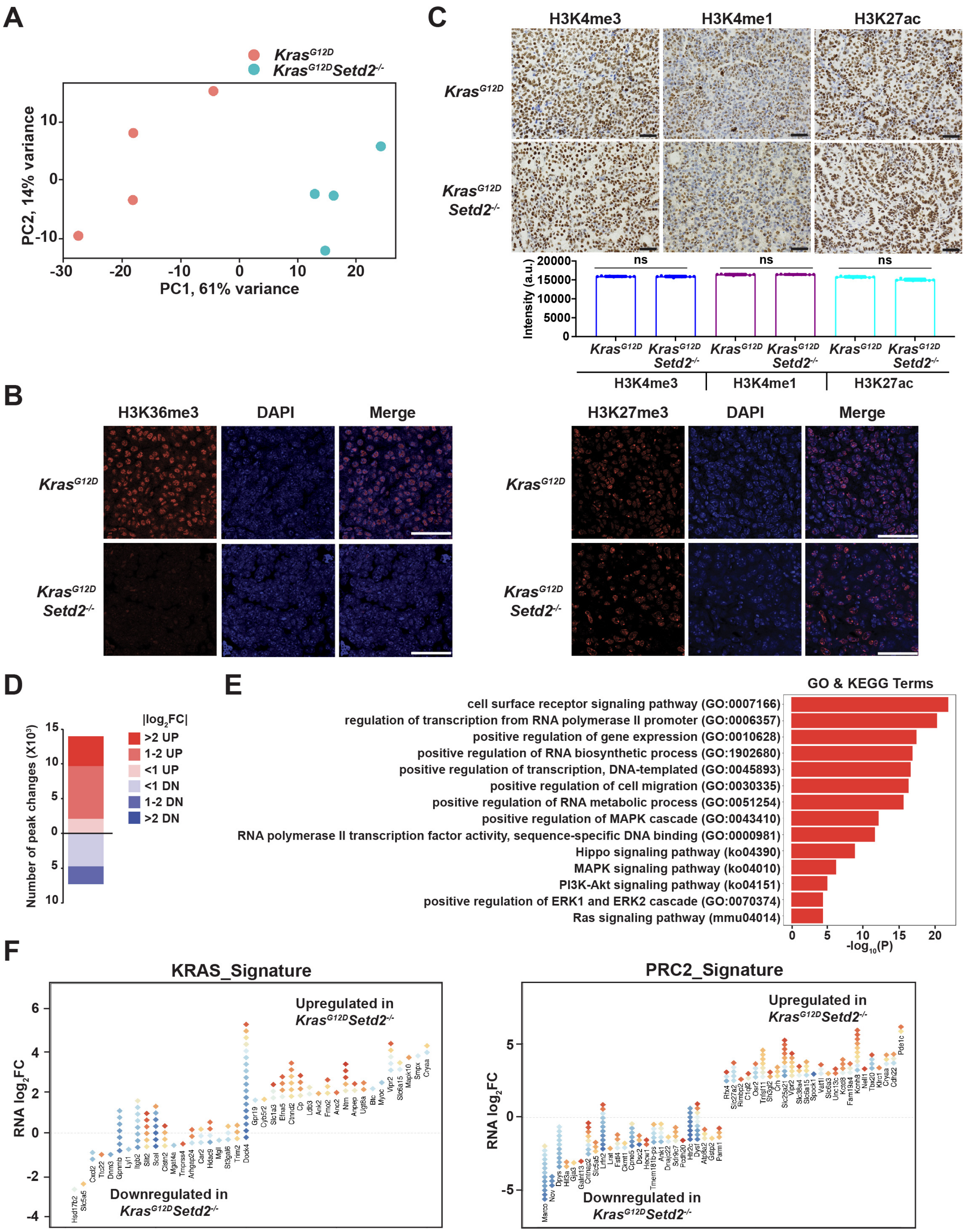
A
KrasG12D
KrasG12D Setd2-/-
PC2, 14% variance
PC1, 61% variance
C
H3K4me3
H3K4me1
H3K27ac
KrasG12D
KrasG12D Setd2-/-
Intensity (a.u.)
ns
B
H3K36me3
DAPI
Merge
H3K27me3
D
Number of peak changes (X10³)
|log2FC|
>2 UP
1-2 UP
<1 UP
<1 DN
1-2 DN
>2 DN
E
GO & KEGG Terms
cell surface receptor signaling pathway (GO:0007166)
regulation of transcription from RNA polymerase II promoter (GO:0006357)
positive regulation of gene expression (GO:0010628)
positive regulation of RNA biosynthetic process (GO:1902680)
positive regulation of transcription, DNA-templated (GO:0045893)
positive regulation of cell migration (GO:0030335)
positive regulation of RNA metabolic process (GO:0051254)
positive regulation of MAPK cascade (GO:0043410)
RNA polymerase II transcription factor activity, sequence-specific DNA binding (GO:0000981)
Hippo signaling pathway (ko04390)
MAPK signaling pathway (ko04010)
PI3K-Akt signaling pathway (ko04151)
positive regulation of ERK1 and ERK2 cascade (GO:0070374)
Ras signaling pathway (mmu04014)
-log10(P)
F
KRAS_Signature
PRC2_Signature
RNA log2FC
Upregulated in KrasG12D Setd2-/-
Downregulated in KrasG12D Setd2-/-

Supplemental Figure 2. (**A**) Principal component analysis (PCA) of RNA-seq expression data of *Kras*^{G12D} and *Kras*^{G12D}*Setd2*$^{-/-}$ mouse lung tumors. (**B**) Representative immunofluorescence images of H3K36me3 and H3K27me3 in *Kras*^{G12D} and *Kras*^{G12D}*Setd2*$^{-/-}$ lung tumors. Scale bars, 50 µm. (**C**) Representative immunohistochemistry (IHC) for H3K4me3, H3K4me1, and H3K27ac in *Kras*^{G12D} and *Kras*^{G12D}*Setd2*$^{-/-}$ lung tumors. Scale bars, 20 µm. IHC intensity (a.u., arbitrary units) was quantified using ImageJ software (mean ± SD, n = 10). ns, not significant (unpaired parametric t test). (**D**) Number of SETD2 loss-induced changes in chromatin accessibility determined by ATAC-seq comparing dissociated *Kras*^{G12D}*Setd2*$^{-/-}$ with *Kras*^{G12D} lung tumor cells. (**E**) The differentially accessible ATAC-seq peaks gained in response to *Setd2* deletion in mouse lung tumors were subjected to Gene Ontology (GO) and KEGG pathway analysis using HOMER. (**F**) Diamond plots of changes in chromatin accessibility for the top 20 most upregulated and 20 most downregulated genes in the KRAS signature as well as the top 25 most upregulated and 25 most downregulated genes in the PRC2 signature in response to *Setd2* deletion. Each gene is illustrated by a stack of diamonds, where each diamond represents a chromatin peak associated with the gene. Red diamonds denote increased or open chromatin accessibility and blue diamonds denote reduced or closed chromatin accessibility in response to *Setd2* deletion.

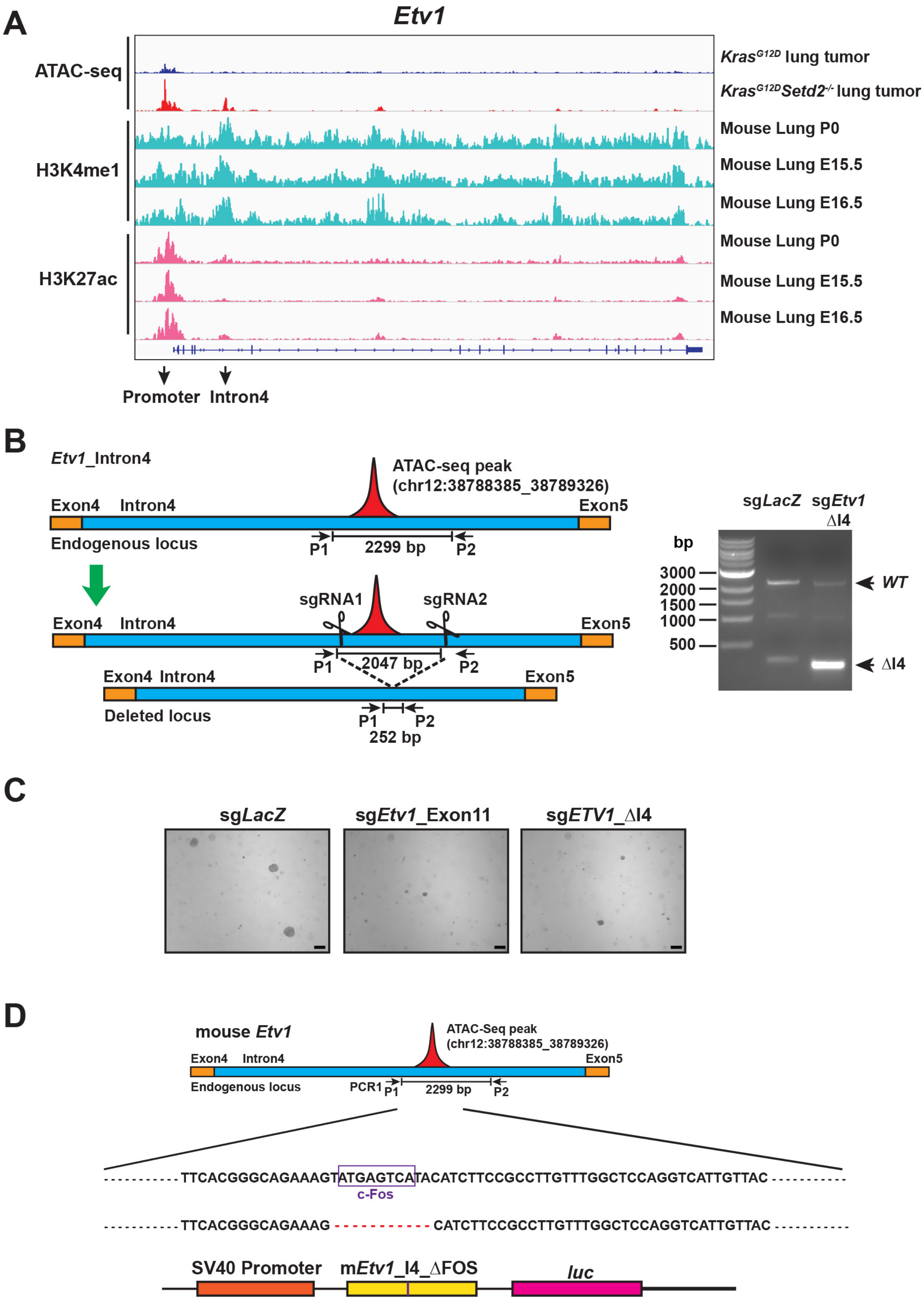
A
Etv1
ATAC-seq
H3K4me1
H3K27ac
KrasG12D lung tumor
KrasG12DSetd2-/- lung tumor
Mouse Lung P0
Mouse Lung E15.5
Mouse Lung E16.5
Mouse Lung P0
Mouse Lung E15.5
Mouse Lung E16.5
Promoter
Intron4
B
Etv1_Intron4
ATAC-seq peak
(chr12:38788385_38789326)
Exon4
Intron4
Exon5
Endogenous locus
P1
2299 bp
P2
sgRNA1
sgRNA2
2047 bp
Deleted locus
252 bp
sgLacZ
sgEtv1 ΔI4
bp
3000
2000
1500
1000
500
WT
ΔI4
C
sgLacZ
sgEtv1_Exon11
sgETV1_ΔI4
D
mouse Etv1
ATAC-Seq peak
(chr12:38788385_38789326)
PCR1
TTCACGGGCAGAAAGTATGAGTCATACATCTTCCGCCTTGTTTGGCTCCAGGTCATTGTTAC
c-Fos
TTCACGGGCAGAAAG CATCTTCCGCCTTGTTTGGCTCCAGGTCATTGTTAC
SV40 Promoter
mEtv1_I4_ΔFOS
luc

Supplemental Figure 3. (**A**) ATAC-seq tracks at the *Etv1* locus in $Kras^{G12D}$ and $Kras^{G12D}Setd2^{-/-}$ mouse lung tumor cells and ENCODE data showing ChIP-seq tracks for H3K4me1 and H3K27ac in mouse lung tissues. (**B**) A schematic diagram of the strategy used to delete the ATAC-seq peak (2047 bp) gained in the intron 4 (9885 bp) of *Etv1* in primary $Kras^{G12D}Setd2^{-/-}$ (KS) mouse lung tumor cells using CRISPR/Cas9-mediated genome editing. The positions of primers (P1 and P2) used for PCR-based validation of genome editing are indicated. PCR-based genotyping using the P1 and P2 primers was performed in KS cells +/- CRISPR/Cas9-mediated deletion of the intron 4 of *Etv1*. (**C**) Representative images of soft agar colony formation assays of primary KS cells infected with lentivirus expressing sgRNAs targeting either *LacZ* or the exon 11 of *Etv1* or KS cells harboring deletion of intron 4 of *Etv1*. Scale bars, 200 µm. (**D**) A schematic diagram of the strategy used to delete the c-Fos binding motif within the ATAC-seq peak region in intron 4 of *Etv1*.

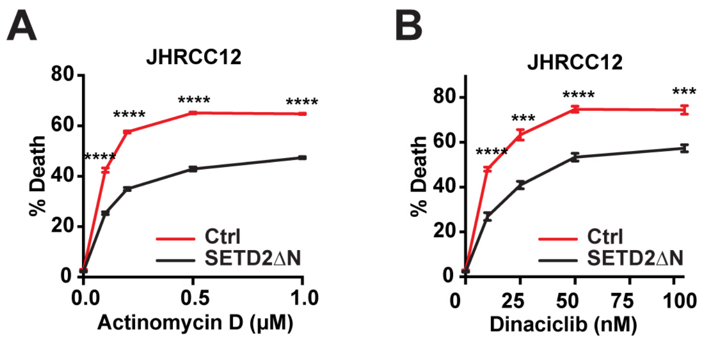

Supplemental Figure 4. JHRCC12 cells infected with control retrovirus or retrovirus expressing SETD2ΔN were treated with actinomycin D (**A**) or dinaciclib (**B**) at the indicated concentrations. Cell death was quantified by annexin-V staining (mean ± SD, n = 3). ***, $P < 0.001$; ****, $P < 0.0001$ (Student's *t*-test).

**NAME**	**NES**	**NOM p-val**	**FDR q-val**
**Signaling**			
PTEN_DN.V1_UP	1.817	0.008	0.011
PTEN_DN.V2_UP	1.783	0.006	0.010
KRAS.300_UP.V1_UP	1.708	0.014	0.018
KRAS.600_UP.V1_UP	1.693	0.014	0.050
KRAS.BREAST_UP.V1_UP	1.589	0.027	0.035
KRAS.KIDNEY_UP.V1_UP	1.589	0.037	0.039
CYCLIN_D1_UP.V1_UP	1.611	0.037	0.035
GO_G_PROTEIN_COUPLED_RECEPTOR_ACTIVITY	1.828	0.000	0.034
GO_REGULATION_OF_G_PROTEIN_COUPLED_RECEPTOR_PROTEIN_SIGNALING_PATHWAY	1.556	0.048	0.048
GO_G_PROTEIN_COUPLED_RECEPTOR_SIGNALING_PATHWAY_COUPLED_TO_CYCLIC_NUCLEOTIDE_SECOND_MESSENGER	1.529	0.039	0.044
GO_SIGNALING_RECEPTOR_ACTIVITY	1.615	0.012	0.016
GO_RECEPTOR_ACTIVITY	1.590	0.007	0.008
GO_CELL_CELL_SIGNALING	1.514	0.019	0.023
**PRC2_target**			
ACEVEDO_LIVER_CANCER_WITH_H3K27ME3_UP	2.190	0.000	0.001
MIKKELSEN_MEF_ICP_WITH_H3K27ME3	1.766	0.010	0.039
MIKKELSEN_NPC_HCP_WITH_H3K4ME3_AND_H3K27ME3	1.730	0.018	0.047
MEISSNER_NPC_HCP_WITH_H3K4ME3_AND_H3K27ME3	1.715	0.021	0.047
BENPORATH_SUZ12_TARGETS	1.662	0.007	0.036
BENPORATH_ES_WITH_H3K27ME3	1.637	0.007	0.006
MEISSNER_NPC_HCP_WITH_H3K4ME2_AND_H3K27ME3	1.519	0.044	0.046
**DNA_packaing**			
GO_DNA_PACKAGING	1.818	0.004	0.033
GO_PROTEIN_DNA_COMPLEX_SUBUNIT_ORGANIZATION	1.996	0.000	0.008
GO_CHROMATIN_ASSEMBLY_OR_DISASSEMBLY	1.790	0.012	0.038
GO_DNA_CONFORMATION_CHANGE	1.772	0.011	0.039
GO_DOUBLE_STRANDED_DNA_BINDING	1.743	0.007	0.045
GO_RIBONUCLEOPROTEIN_COMPLEX	1.545	0.034	0.050
**RNA catabolism**			
GO_NUCLEAR_TRANSCRIBED_MRNA_CATABOLIC_PROCESS_NONSENSE_MEDIATED_DECAY	2.691	0.000	0.000
REACTOME_NONSENSE_MEDIATED_DECAY_ENHANCED_BY_THE_EXON_JUNCTION_COMPLEX	2.680	0.000	0.000

GO_RNA_CATABOLIC_PROCESS	2.603	0.000	0.000
REACTOME_METABOLISM_OF_MRNA	2.514	0.000	0.000
REACTOME_METABOLISM_OF_RNA	2.426	0.000	0.000
GO_MRNA_METABOLIC_PROCESS	2.386	0.000	0.000
GO_RRNA_METABOLIC_PROCESS	2.117	0.000	0.002
GO_POSITIVE_REGULATION_OF_NUCLEOTIDE_METABOLIC_PROCESS	1.704	0.029	0.048

Supplemental Table 1. Summary of GSEA of the differentially expressed genes (FDR < 0.05) detected by RNA-seq comparing $Kras^{G12D}Setd2^{-/-}$ with $Kras^{G12D}$ lung tumors.
